# Experimental Analysis of HPV16 L1/L2 Chimeric VLP Internalization by Human Peripheral Blood Leukocytes

**DOI:** 10.3390/ijms27156968

**Published:** 2026-08-03

**Authors:** Aurora Marques Cianciarullo, Dirce Sakauchi, Erica Akemi Kavati Sasaki, Tania Matiko Hosoda, Primavera Borelli, Willy Beçak

**Affiliations:** 1 Laboratory of Genetics, Butantan Institute, Secretary of Sao Paulo State for Health, 1500 Dr. Vital Brazil Avenue, Sao Paulo 05503-900, Brazil; dirce.sakauchi@butantan.gov.br (D.S.); eakavati@yahoo.com.br (E.A.K.S.); hosodatania@yahoo.com.br (T.M.H.);; 2 Program of Postgraduate Interunit in Biotechnology USP-IBU-IPT, Institute of Biomedical Sciences, University of Sao Paulo, 2415 Prof Lineu Prestes Avenue, University City, Sao Paulo 05508-900, Brazil; 3 Program of Professional Upgrading at Butantan Institute, Secretary of Sao Paulo State for Health, 188 Dr. Eneas de Carvalho Aguiar, Sao Paulo 05403-000, Brazil; 4 Laboratory of Experimental and Clinical Hematology, Department of Clinical and Toxicological Analyses, School of Pharmaceutical Sciences, University of Sao Paulo, 580 B17 Prof Lineu Prestes Avenue, University City, Sao Paulo 05508-900, Brazil; borelli@usp.br

**Keywords:** HPV16 L1/L2 chimeric VLPs, molecular engineering, VLP-host leukocyte internalization, chimeric VLP uptake by lymphocytes, membrane receptors, human leukocytes, mononuclear cells, cancer risk factors, infectious diseases, immunity, molecular virology

## Abstract

Human papillomavirus type 16 (HPV16) is a major etiological agent of cervical and other epithelial cancers, yet the mechanisms underlying host–pathogen interactions remain incompletely understood. In this study, we investigated the responses of human peripheral blood leukocytes to engineered HPV16 L1/L2 chimeric virus-like particles (VLPs), produced in suspension by HEK 293-F cells. These VLPs were designed to mimic native viral structures while incorporating chimeric features that enhance stability and immunogenicity. Through experimental assays, we characterized leukocyte engagement, primarily involving leukocyte phenotyping, VLP internalization, confocal colocalization, and endocytic pathway analyses. We demonstrated that recombinant L1/L2 proteins assembled into structured VLPs capable of interacting with mononuclear cells, including lymphocytes and monocytes, but not with polymorphonuclear cells, such as neutrophils, eosinophils and basophils. Uptake occurred via the CD71 transferrin receptor-mediated pathway, in addition to other endocytic routes analyzed, as confirmed by blockage assays using chlorpromazine, rCTB, filipin, nystatin, liquemine, and sodium azide. Confocal colocalization and endocytic pathway analyses further supported receptor-mediated uptake. These findings demonstrate that HPV16 L1/L2 chimeric VLPs interact with and are internalized by human peripheral blood mononuclear cells through CD71-associated and other endocytic pathways. The study provides new insights into HPV16 VLP–leukocyte interactions and contributes to a better understanding of the cellular mechanisms involved in VLP uptake, which may be relevant for future studies on HPV biology and VLP-based vaccine development.

## 1. Introduction

Harald zur Hausen’s pioneering research established the etiological significance of human papillomavirus (HPV) in cervical carcinogenesis, identifying HPV as the principal causal agent. Subsequent investigations corroborated these findings, demonstrating the presence of HPV DNA in neoplasms arising in other anogenital sites [1,2,3]. To date, over 200 HPV types have been characterized, of which approximately 40 types are known to infect the genital tract. Within this group, HPV types 6 and 11 are predominantly associated with benign proliferative lesions such as genital warts, whereas HPV types 16 and 18 exhibit strong oncogenic potential, being consistently implicated in malignant transformation and cancer development. According to the International Agency for Research on Cancer (IARC), at least 12 HPV types are classified as oncogenic in humans [4]. Comprehensive genomic and epidemiological data are available through resources such as the Papillomavirus Episteme (PaVE). Among them, HPV16 and HPV18 are responsible for approximately 70% of all cervical cancers [4,5]. In 2025, the projected global estimate of cervical cancer is expected to rise to 720,415 new cases per year, and it is expected that half of these women will die from this neoplasia [5]. Novel HPV lineages and sub-lineages have been described in HIV/HPV-co-infected pregnant women, with two high-risk types [6]. However, the difficulty in obtaining enough viable wild types or recombinant HPV particles has limited research to distinct aspects of virus biology [7].

Viruses depend on entry into host cells to ensure their survival, replication and ability to evade the immune system’s defense mechanisms. This initial infection process is fundamental to the viral cycle, as it allows the pathogen to use the host’s cellular machinery to synthesize viral proteins, replicate its genetic material and produce new infectious particles. Furthermore, the ability to escape immunological surveillance is a determining factor for the persistence of infection and the establishment of associated diseases [8]. A detailed understanding of these viral strategies is essential for the development of effective therapeutic interventions and vaccines. So far, the entry of HPV and its trafficking by the host cell are still not fully elucidated [9,10,11]. There is evidence that BPV1, HPV16 and HPV58 are internalized in a clathrin-mediated manner, while HPV31 can use caveolin- or clathrin-dependent endocytosis, suggesting that the entry of HPV into the host cell can be a complex process [12,13]. 

In this context, virus-like particles composed of L1 and L2 capsid proteins without a viral genome were expressed in human epithelial kidney cell line 293-T and 293-F cultures, using the vectors pUF3/L1h and pUF3/L2h (Figure 1), constructed under the regulation of the promoter of human cytomegalovirus for pathogen–host interaction assays [14,15,16,17,18,19,20].

HPV L1 and L2 proteins are difficult to express in mammalian cells, limiting DNA vaccine efficacy. By optimizing the coding sequence—especially codon usage—it was achieved robust expression of HPV16 L1 and L2, leading to abundant virus-like particle formation. Codon-adapted L1 constructs generated markedly stronger immune responses in vivo, underscoring codon optimization as a powerful strategy for enhancing HPV DNA vaccine performance and increase its potential applications. Transient expression of HPV16 L1 and L2 proteins can be substantially enhanced by optimizing the RNA coding sequence [14].

The pUF3L1h and pUF3L2h gene expression vectors are circular maps illustrating the modular architecture of both plasmids, each employing the CMV promoter to drive strong transient expression in mammalian cells. This design enables flexible expression of engineered PUF RNA-binding proteins under diverse experimental conditions, broadening their potential applications (Figure 2).

Evidence suggests that the process of mechanisms governing HPV entry into host cells involves multiple steps of interaction between viral proteins and cell membrane components, in addition to the use of specific endocytic pathways to facilitate internalization. However, the complexity of these interactions, combined with the diversity of viral subtypes and variations in the cellular microenvironment, makes detailed characterization of these events difficult. An in-depth understanding of these mechanisms is essential for the development of more effective therapeutic and preventive strategies against persistent infections and their potential oncogenic consequences [7,8,11].

Human papillomavirus is a small, non-enveloped, double-stranded DNA virus with a tropism for squamous epithelium. HPV is approximately 60 nanometers in diameter and is made up of a highly organized icosahedral capsid, which is responsible for protecting and transporting the viral genome. This genome consists of a circular double-stranded DNA molecule with around 8000 base pairs, whose compaction reflects the evolutionary efficiency of the virus. Interestingly, only one of the DNA strands is used as a template for transcription, which confers specificity to the gene expression process. The genome is functionally divided into three main regions: the early region, which encodes proteins involved in viral replication and modulation of the host response; the late region, responsible for the synthesis of capsid structural proteins; and the regulatory or non-coding region, which contains elements essential for the control of transcription and replication. In total, ten open reading frames (ORFs) are identified, and their coordinated expression guarantees the orderly progression of the viral cycle. This compact and functional genomic architecture is fundamental to the ability of HPV to establish persistent infections and contribute to pathological processes, including oncogenesis in epithelial tissues [10,11,21].

The life cycle of HPV is intimately dependent on the cellular differentiation of the squamous epithelium and reflects a highly specialized evolutionary strategy. Infection begins in the basal cells of the epidermis, usually after microlesions that allow viral access. Once internalized, the circular viral DNA is maintained as an episome in the nucleus, ensuring the persistence of the infection without causing immediate lysis of the host cell. As infected cells migrate to suprabasal layers, HPV intensifies the replication of its genome and coordinates the expression of early genes responsible for regulating replication and modulating the immune response. In the more superficial and differentiated layers, the expression of late genes that encode the structural proteins of the capsid occurs, culminating in the assembly of new virions. Viral release coincides with the natural process of epithelial cell desquamation, allowing efficient dissemination without the need for cell lysis, known as productive infection. In oncogenic subtypes, the integration of viral DNA into the host genome can result in the continuous expression of E6 and E7 oncoproteins, which inactivate p53 and pRb, promote genomic instability and increase the risk of malignant transformation, termed transforming infection. Persistent infection can result in epithelial hyperplasia, warts, and, in some cases, progression to precancerous lesions. Thus, the HPV life cycle not only ensures its perpetuation but also lays the groundwork for its clinical relevance and oncogenic potential [9,10,21].

HPV L1 virus-like particle vaccines, traditionally administered in three doses over six months, have demonstrated strong prophylactic efficacy in clinical trials and national immunization programs. Emerging evidence suggests that a single dose may confer comparable protection, likely owing to the structural properties of VLPs that elicit durable antibody responses and the unique HPV life cycle, which renders virions particularly susceptible to antibody-mediated inhibition [22].

Primary HPV testing is the most effective and cost-efficient cervical cancer screening strategy, reducing mortality by over 63% when performed every 5 years with adequate coverage. Triage of HPV-positive women via genotyping (HPV DNA detection), cytology (Papanicolaou test), VIA (visual inspection with acetic acid), or colposcopy (magnifying instrument for biopsy) achieves similar outcomes while limiting overtreatment. In contrast, VIA or cytology alone are less effective and generate more unnecessary procedures. Even limited HPV testing—twice in a lifetime—remains impactful, lowering mortality by at least 41% [23].

Nonetheless, a wide range of laboratory-based assays for the detection of HPV are employed across clinical, epidemiological, and research contexts. These assays utilize diverse biological specimens, which may be collected either by healthcare professionals or through self-sampling procedures performed by the individuals themselves. In addition to conventional cervical and cervicovaginal samples, several alternative specimen types—such as urine, blood, and oral samples—have been investigated for HPV testing [5,6,7,24].

Among these, first-void (first morning) urine has emerged as a particularly promising specimen. Evidence increasingly supports its reliability for HPV-based cervical cancer screening, follow-up testing in women with HPV-positive results, monitoring the population-level impact of HPV vaccination programs, and facilitating HPV detection in cohorts for whom less invasive sampling methods are preferable. The methodological advantages of urine sampling include its non-invasive nature, ease of collection, and potential for large-scale implementation in screening programs. However, limitations remain, such as variability in viral load across different specimen types, the need for standardized protocols for sample processing, and the requirement for further validation in diverse populations [5,6,7,23,24,25].

Taken together, the incorporation of urine and other alternative samples into HPV testing strategies represents a significant advancement toward more accessible, acceptable, and scalable approaches to cervical cancer prevention and HPV surveillance. The unceasing expression of the HPV oncoproteins E6 and E7 is essential for the transformation and maintenance of cancer cells. For this reason, therapeutic targeting of E6 or E7 oncogenes can potentially treat HPV-related cancers. Some studies have revealed that besides the sexual transmission of HPV, there are other forms of infection. A molecular epidemiology field study conducted in India assessed the prevalence, incidence, and dynamics of oral human papillomavirus infection in healthy individuals. Demographic, behavioral, and oral gargle samples were collected for biochemical and molecular analyses. HPV DNA was detected in 284 (98%) participants, with HPV16 being the most common, alongside isolated detections of HPV18, HPV66, HPV70, and HPV89. Ten novel variants were identified—nine of HPV16 and one of HPV89—while the remaining sequences aligned with already established Indian cervical HPV lineages [26].

Alterations in DNA can compromise cellular repair mechanisms, activate oncogenes, or inactivate tumor suppressor genes, while external factors—such as exposure to carcinogenic agents, lifestyle habits, and environmental conditions—contribute to increasing the risk of developing neoplasms. The interaction between genetic predisposition and environmental influences highlights the multifactorial complexity of cancer and reinforces the need for integrated approaches to its prevention and treatment [10,11,27].

The tumor immune microenvironment (TIME) plays a pivotal role in shaping the pathogenesis and therapeutic responsiveness of cervical cancer. Current understanding encompasses the complex cellular and molecular architecture of the TIME, including patterns of immune cell infiltration—such as T cells, B cells, natural killer (NK) cells, dendritic cells, and tumor-associated macrophages—alongside immune checkpoint regulation and cytokine–chemokine signaling networks. Advances in high-resolution spatial profiling and single-cell sequencing have unveiled remarkable heterogeneity within the TIME, highlighting its dynamic evolution across disease stages and treatment contexts. Emerging immunotherapeutic approaches, including checkpoint blockade, adoptive cell transfer, and therapeutic vaccination, continue to expand the clinical landscape, emphasizing their underlying mechanisms, efficacy, and translational challenges [28].

Furthermore, the HPV life cycle is non-lytic, avoiding the release of danger signals associated with cell death and consequently reducing the activation of the innate immune response. The virus also modulates the expression of cytokines and antigen-presenting molecules, such as MHC class I, decreasing the ability of infected cells to be recognized by cytotoxic T lymphocytes [29,30].

Early viral proteins, particularly E6 and E7, play a central role in immune evasion. In addition to inactivating p53 and pRb to promote viral replication and cell transformation, these oncoproteins interfere with interferon signaling and dendritic cell activity, compromising the induction of effective antiviral responses. HPV also reduces the expression of ligands for NK cell activating receptors, limiting the action of these cells in eliminating infected cells. This coordinated suppression of innate immunity directly impacts the adaptive response, hindering the generation of effector and memory T lymphocytes [11,17,19,31].

The persistence of HPV, especially in oncogenic subtypes, is associated with a state of local and systemic immunosuppression. In cervical tissues, for example, the formation of a tolerogenic immunological microenvironment is observed, characterized by an increase in regulatory T cells and the secretion of immunosuppressive cytokines, such as IL-10 and TGF-β. This scenario favors the maintenance of chronic infection and contributes to the progression of pre-malignant lesions to invasive cancer. A detailed understanding of these interactions between HPV and the immune system is crucial for the development of therapeutic vaccines and targeted immunotherapies capable of restoring immune surveillance and eliminating infected or transformed cells [10,19,32].

Despite evasion strategies, the immune system can control HPV infection in many cases. Cellular immunity, especially mediated by CD8+ T lymphocytes, plays a crucial role in eliminating infected cells. The humoral response also contributes, with neutralizing antibodies against L1 and L2 capsid proteins, although its effectiveness is limited in established infections. Viral persistence, however, reflects the insufficiency of these responses in oncogenic subtypes, favoring progression to pre-malignant lesions and cancer [8,33].

In addition, modern immunotherapies, such as immune checkpoint inhibitors (anti-PD-1/PD-L1), have shown potential in restoring immune surveillance in patients with advanced cervical cancer. The combination of therapeutic vaccines with immunomodulators represents a promising approach to overcoming HPV immune evasion. HPV-induced cancers represent an approaching risk, and there potential for CRISPR/Cas9 in targeted therapy [34,35,36,37,38,39]. Unlike prophylactic vaccines, therapeutic vaccines aim to induce a cellular response against early proteins, such as E6 and E7, in order to eliminate already infected or transformed cells [36]. Strategies include DNA, RNA, peptide, and viral vector-based vaccines [37].

The interaction between HPV and the immune system is marked by a sophisticated network of viral evasion and incomplete immune responses. While prophylactic vaccines have revolutionized prevention, the current challenge lies in developing therapeutic strategies capable of eliminating persistent infections and preventing tumor progression [37,40,41].

Several HPV genes actively modulate host immunity to promote viral persistence and oncogenesis. The best-characterized immunomodulatory genes are E6, E7, and E2, which interfere with innate and adaptive immune signaling, antigen presentation, and cytokine production [42]. In summary, HPV’s E6, E7, E2, and E5 genes orchestrate a multifaceted immune evasion strategy, dampening both innate and adaptive responses and enabling persistent infection and malignant transformation.

High-risk (HR) types, such as HPV16 and HPV18, are strongly associated with cervical and other anogenital cancers, whereas low-risk (LR) types, including HPV11, are primarily linked to benign conditions. Although the immune evasion mechanisms mediated by HPV oncoproteins E6 and E7 have been extensively characterized, the immunomodulatory functions of the E2 protein remain comparatively underexplored [42].

The role of HPV11 and HPV16 E2 proteins in modulating innate antiviral immunity, with emphasis on their interactions with key signaling pathways, has been demonstrated. E2 proteins from both HPV types effectively suppress the activation of critical antiviral cascades, including RIG-I/MDA5–MAVS, TLR3–TRIF, cGAS–STING, and JAK–STAT. Mechanistic analyses reveal that E2 interacts with core components of type I interferon (IFN)-inducing pathways, inhibiting IRF3 phosphorylation and nuclear translocation, thereby attenuating IFN expression. Furthermore, E2 disrupts the JAK–STAT signaling axis by preventing the assembly of the ISGF3 complex (STAT1–STAT2–IRF9), ultimately suppressing the transcription of interferon-stimulated genes (ISGs) [42].

Collectively, these findings highlight the broad immunosuppressive capacity of HPV E2 proteins, complementing the well-established immune evasion strategies mediated by E6 and E7. This concept advances the understanding of HPV-driven immune modulation and identifies E2 as a potential therapeutic target for enhancing antiviral immunity in HPV-associated diseases [42].

The state of the art is presented here with an emphasis on the remarkable advances achieved in research within the complex universe of HPV. Over the past decades, scientific efforts have progressively unveiled the molecular mechanisms underlying HPV infection, replication, and pathogenesis, providing crucial insights into viral tropism, immune evasion, and oncogenic potential. These advances have not only deepened our understanding of HPV biology but have also paved the way for innovative diagnostic tools, therapeutic approaches, and preventive strategies.

Whereas new pathologies are increasingly being associated with HPVs, the responsibility and costs of HPV-associated diseases and cancer remain an important public health issue in all countries, regardless of their economic developmental level [19,37,43]. Due to the continuous worldwide propagation of HPV, it is necessary to investigate the possibility of HPV crosstalk among different human cell types. In the present study, we addressed the capacity of HPV16 L1/L2 chimeric VLP crosstalk between human peripheral blood leukocytes ex vivo.

## 2. Results

### 2.1. Leukocyte Identification

After isolating leukocytes from healthy adult female donors, control smears stained with the May–Grünwald–Giemsa method demonstrated the preservation of cell morphology (Figure 3), showing a positive correlation with control blood smears. Cell counts indicated approximately 98% lymphocytes, 0.7% monocytes, 1.5% polymorphonuclear cells, 0.1% red blood cells, and 0.1% platelets. Cell viability was confirmed by Rhodamine 123 mitochondrial labeling, a specific marker for living cells (Figure 4). The ultrastructure was well preserved across all cell types analyzed, with intact and well-defined membranes (Figure 5). The efficiency and rapidity of the method in obtaining leukocytes, while maintaining morphology and cell viability, were confirmed.

### 2.2. Expression and Localization of Recombinant Proteins HPV16 L1 and L2 Capsid Proteins in HEK 293-T and HEK 293-F Cells

The established HEK 293-T cell line grows attached, while the HEK 293-F cell line was developed for suspension cultivation, suitable for use in bioreactors for large-scale production (Figure 6). Both proteins were detected intracellularly in the nucleus and cytoplasm of both cell lines. The presence of these heterologous proteins, as well as the formation of virus-like particles (VLPs), was confirmed by transmission electron microscopy (TEM) through colloidal gold immunolabeling and negative staining (Figure 7). Cell extracts containing recombinant proteins were purified by affinity chromatography, and the immunization of Balb/c mice with HPV16 L1/L2 VLPs formulated with adjuvant induced higher titers of anti-HPV16 L1 antibodies compared to HPV16 L2, as determined by indirect ELISA. These findings demonstrate that transient expression in HEK 293-T and 293-F cells was efficiently achieved. The results are promising for the production of recombinant HPV capsid proteins for future studies on human papillomavirus and may contribute to the development of novel vaccine strategies for HPV prevention [16,17,18,20].

### 2.3. Analysis of HPV16 L1/L2 Chimeric VLP Crosstalk by HEK 293-T Cells

The uptake of HPV16 L1/L2 virus-like particles (VLPs) by HEK 293-T cells was analyzed using immunofluorescence (Figure 8). Notably, the genome of this cell line does not contain HPV DNA sequences, but does harbor adenovirus DNA and SV40 T-Ag. After 4 h of incubation at 37 °C, the cells were thoroughly washed to remove non-internalized particles and proteins, then fixed and immunostained. Detection was performed using the Camvir-1 monoclonal antibody (BD Biosciences; Table 1), which is specific for L1 proteins, together with an L1 conformation-specific anti-VLP antiserum (Biodesign; Table 1).

During the purification of HPV16 L1 and L2 proteins, clarified cell lysates were obtained from HEK293-F cells cotransfected with the L1 (pUF3L1h) and L2 (pUF3L2h) expression vectors. The resulting HPV16 L1/L2 cell extract was lysed, clarified, and subjected to ammonium sulfate precipitation to remove contaminants. The sample was then dialyzed and further purified by size-exclusion or affinity chromatography.

The purity of HPV16 L1/L2 clarified cell lysate samples and the fractions eluted from size-exclusion chromatography were analyzed by 10% SDS-PAGE under reducing conditions (Figure 9 and Figure 10). The electrophoretic profile of the clarified lysate displayed a prominent band at approximately 55 kDa, consistent with the molecular mass of the L1 protein, and a band near 72 kDa, corresponding to the L2 protein. Additional bands were observed around 50 kDa, 60 kDa, and 100 kDa (Figure 9, lane 2). The eluted fraction from chromatography revealed bands at ~50 kDa, 60 kDa, 72 kDa, 120 kDa, and 140 kDa (Figure 9, lane 3). In eluted fraction 2, bands at ~50 kDa and 72 kDa were detected (Figure 9, lane 4), while fraction 3 similarly exhibited bands at ~50 kDa and 72 kDa (Figure 10, lane 5).

Western blotting was used to analyze intracellular expression of HPV16 L1 and L2 proteins. Using the anti-L1 monoclonal antibody (Camvir-1), a ~55 kDa band corresponding to L1 was detected in the cleared HPV16 L1/L2 lysate and in purified fractions 1–3, while no signal was observed in non-transfected HEK 293-F controls. Similarly, analysis with an anti-L2 polyclonal antibody revealed a ~72 kDa band consistent with L2 in the lysate and chromatography fractions, along with additional immunoreactive bands (~40–100 kDa). Non-transfected controls showed nonspecific bands at ~50–60 kDa. Overall, both L1 and L2 proteins were expressed and specifically recognized, confirming effective production and purification of HPV16 L1/L2 chimeric VLP components.

To assess the expression kinetics of HPV16 L1 and L2 proteins, HEK293-T and HEK293-F cells were transiently cotransfected with pUF3/L1h and pUF3/L2h vectors. Cells were analyzed at 6, 12, 24, 36, and 48 h post-transfection by confocal microscopy and indirect immunofluorescence using anti-HPV16 L1 (monoclonal) and anti-HPV16 L2 (polyclonal) antibodies. Both proteins were detected in all samples, with peak expression at 48 h (Figure 11A’,B’). Flow cytometry confirmed increasing A and B expression from 12 to 36 h, followed by a slight decrease at 48 h. Fluorescence intensity analysis showed maximal signals for both L1 and L2 at 48 h. Based on these results, subsequent experiments were conducted at 48 h post-cotransfection in HEK 293-F cells to evaluate HPV16 L1/L2 protein expression.

**A.**  The cells were transfected with pUF3/L1h and pUF3/L2h vectors (Figure 1 and Figure 2) and, after 48 h, fixed with 2% PFA in PBS. In (a), the cells were treated with an anti-L1 antibody, and in (b) with a conformational anti-VLP antibody, both for HPV16, and subsequently revealed with a secondary antibody conjugated to FITC (green). In both assays, the actin cytoskeleton detected by Phalloidin conjugated with AlexaFluor^®^ 594 was labeled (red). Control assays for detection of HPV16 L1 and VLPs produced in HEK 293-T cells by indirect immunofluorescence: In (a), the cells were immunolabeled with an anti-L1 antibody and in (b) with a conformational anti-VLP antibody, both for HPV16, and revealed with a FITC-conjugated secondary antibody (green). In both assays, the actin cytoskeleton was detected by Phalloidin conjugated with AlexaFluor^®^ 594 (red). CLSM Zeiss LSM 510 Meta. Magnification: 63× C-Apochromatic objective /1.4 Oil. Scale bar = 10 µm.
**B.**  Kinetics of L1 protein expression in 293-F cells transfected with the pUF3/L1h vector, analyzed by confocal microscopy and flow cytometry. The cells were examined at different post-transfection time points (6–72 h). (a) Merged image of panels B and C. (b) L1 protein detected with a monoclonal anti-HPV16L1 antibody, revealed using goat anti-mouse IgG conjugated to AlexaFluor^®^ 488 (green). (c) Transmitted light channel (DIC). CLSM Zeiss LSM 510 Meta. Magnification: 63× C-Apochromatic objective/1.4 Oil. Scale bar = 10 µm. In the same way, samples analyzed by flow cytometry showed high expression of these recombinant proteins. Cells expressed the L1 and L2 proteins between 24 and 48 h of cellular transfection, corroborating with data obtained by CLSM.

Interactions between HEK 293-T cells and HPV16 L1/L2 chimeric VLPs were analyzed by immunofluorescence. Non-transfected HEK 293-T cells, which lack papillomavirus sequences, were incubated with VLPs for 4 h at 37 °C, washed, fixed, and labeled with anti-L1 (Camvir-1) and conformational anti-VLP antibodies. Internalized VLPs were observed in the cytoplasm near the nucleus (Figure 12A–E), confirmed by overlay images and Z-axis scans. In cases with higher particle uptake, endocytic vesicle-like structures were detected. Similar distribution was observed with the L1 capsid antibody. Both assembled VLPs and L1 monomeric/pentameric forms crossed the cell membrane after 4 h. Negative controls showed no immunostaining or morphological changes (Figure 12F,G).

### 2.4. Analysis of HPV16 L1/L2 Chimeric VLP Crosstalk by Human Leukocytes

HPV16 L1/L2 virus-like particles (VLPs) were incubated with human leukocytes for 4 h at 37 °C and subsequently prepared for indirect CLSM immunofluorescence analysis to assess their potential internalization. The results demonstrated the uptake of VLPs by peripheral blood mononuclear cells (PBMCs), including T and B lymphocytes and monocytes, from healthy female volunteers (Figure 13). Structured HPV16 L1/L2 VLPs interacted with and were internalized by leukocytes (Figure 14 and Figure 15).

After 4 h of incubation, VLPs were predominantly observed within the cytoplasm of most leukocytes examined, similar to the findings in HEK 293-T cells. These observations were confirmed by image overlays (Figure 13A,B,D, Figure 14 and Figure 15) and by Z-axis scanning sections that revealed detailed internalization (Figure 13C). Figure 13C (green arrow) highlights VLPs crossing the cell membrane. Z-axis sweeps further demonstrated internalization, with larger numbers of particles forming endocytic vesicle-like structures (Figure 14A, white arrow), comparable to those seen in HEK 293-T cells, a phenomenon supported by morphological evaluations.

### 2.5. Identification of VLP Internalization by Human PBMCs

Peripheral blood mononuclear cells (PBMCs) were treated with antibodies specific to cell membrane receptors (Table 1 and Table 2) and analyzed by indirect immunofluorescence using CLSM to assess interactions with HPV16 VLPs after 4 h of incubation at 37 °C. HPV16 VLPs interacted with ex vivo PBMCs (Figure 16, Figure 17 and Figure 18). These findings were confirmed by image overlays (Figure 16 and Figure 17) and by detailed Z-axis sectioning, which revealed internalization (Figure 18).

T and B lymphocytes exhibited greater competence for VLP uptake, with approximately 47–52% of cells internalizing particles (Table 3). Morphological assessments showed that T lymphocytes positive for anti-CD8 and internalizing VLPs displayed endocytic vesicle-like structures (Figure 16B, white arrow). In contrast, only 23% of monocytes identified with the anti-CD14 antibody interacted with VLPs (Table 3).

### 2.6. Analysis of Colocalization of VLPs and Transferrin (Tf) in Human PBMCs

After 15 min of incubation, no colocalization between L1 VLPs and exogenous transferrin (Tf) was detected (Figure 19). However, the internalization of VLPs was already evident at this time. By 45 min, colocalization of VLPs with exogenous Tf in the cytoplasm of human PBMCs (Figure 19 and Figure 20, yellow arrows), as well as with the transferrin receptor (TfR/CD71), was observed (Figure 20A,B, yellow arrows), suggesting that HPV internalization may occur via the iron uptake pathway. These findings were confirmed by image overlays (Figure 19 and Figure 20).

We compared the kinetics of Tf and TfR (CD71) internalization with the colocalization of HPV16 L1/L2 VLPs produced in this study and control HPV6, 11, 16, and 18 L1 VLPs derived from the Gardasil^®^ vaccine (Figure 20). After only 15 min of incubation, colocalization of TfR and Tf (Figure 20 A,B, purple arrows) was observed with both L1/L2 VLPs (Figure 20A) and Gardasil L1 VLPs (Figure 20B). Morphological analysis further demonstrated colocalization of both L1 and L1/L2 VLPs with Tf and TfR in the cytoplasm of leukocytes after 45 min (Figure 20 C,D, white arrows). This colocalization was detected in approximately 50% of the analyzed cells.

At 60 min, weak colocalization between Tf and VLPs was observed in about 30% of cells (Figure 20E,F, yellow arrows), while no colocalization was detected after 120 min. Three-dimensional Z-axis reconstructions confirmed these observations (Figure 20). Negative controls showed no immunostaining for L1 or VLPs, although exogenous Tf internalization was evident. Comparative analyses between L1/L2 VLPs produced in this study and Gardasil^®^ L1 VLPs revealed no significant differences in the results.

### 2.7. Analysis of the Blocking Assays of PBMC Membrane Receptors

After positive identification of VLPs within leukocytes and their colocalization with Tf and TfR, a variety of biochemical inhibitors (Table 4), known to inhibit distinct cellular processes, were used to demonstrate the involvement of these and other different pathways of VLP entry in PBMC. Chlorpromazine inhibits clathrin-mediated endocytosis of various plasma membrane proteins. Nystatin is a sterol-binding agent that disassembles caveolae in the membrane. rCTB (Clostridium toxin B) acts by shortening the length of actin filaments, by inhibiting this process in vitro. Liquemine is related to the obstruction of VLP internalization through the heparan sulphate proteoglycans (HSPG) pathway. Sodium azide is an ATPase inhibitor that blocks particle movement towards the cell body and leads to diffuse random movement. The use of these different biochemical inhibitors was not sufficient to block HPV16 L1/L2 VLP entry in PBMC (Figure 21 and Figure 22). Chlorpromazine, although known for blocking the clathrin pathway through the CD71 receptor of Tf, was not able to inhibit VLP entry into PBMC in a satisfactory manner. The same occurred when rCTB, Filipin, Nystatin, Liquemine and sodium azide were used to block actin-dependent cell processes, the caveolae pathway, the HSPG pathway, and endocytic pathways, respectively, either assayed separately or combined in an all-inhibitor cocktail. These results suggest that PBMC makes use of multiple internalization pathways to uptake HPV16 L1/L2 VLPs.

## 3. Discussion

Unlike many Beta and Gamma papillomaviruses, Alpha papillomaviruses have developed immune system evasion strategies from the host, causing persistent and visible squamous papillomas, which sometimes evolve into cancers [44,45,46]. Some HPV types, including 16, 18, 31, 33, 35, 39, 45, 51, 52, 56, 58 and 59, have been classified as human carcinogens by the IARC, being responsible for at least 4% of all human malignancies [4,46,47]. Infection by high-risk HPV types is the major factor in the development of human cervical cancer, and HPV DNA is also found in tumors affecting other anogenital regions. Furthermore, they are being correlated with an increasing proportion of squamous cell carcinomas of the oropharynx, with occurrences primarily affecting the tonsils and base of the tongue documented in specific geographic areas [48,49,50]. Cell–virus interaction is the initial step in viral infection. Often, virus adsorption occurs on the plasma membrane of susceptible cells. In general, the most common solution is that pathogen intracellular trafficking occurs through an existing entry mechanism, mediated via clathrin, caveolin, macropinocytosis, among others, moving inside cells to reach the location where viral replication occurs [51]. Transcytosis is used to move antigens and protective antibodies across epithelial barriers. Similar to what seems to occur with HIV [52], the transcytosis of HPV through epithelial cells, crossing cellular barriers, should also be dependent on trafficking to the endocytic recycling pathway. This means that the route by which internalized molecules (like receptors, lipids, or proteins) are returned from endosomes back to the plasma membrane instead of being degraded in lysosomes. It is a critical mechanism for maintaining membrane composition, receptor availability, and overall cellular homeostasis [51,52].

The productive life cycle of HPV is intimately coupled with the process of epithelial cell differentiation. As basal cells divide and mature, the virus exploits this differentiation program to regulate its replication and gene expression. Structural genes, particularly L1 and L2, are expressed in the upper epithelial layers under the strict control of viral transcriptional and translational regulatory mechanisms. This finely tuned regulation ensures that capsid proteins are produced only when the host cell environment is favorable for virion assembly and release, thereby linking viral propagation directly to epithelial maturation [53]. During HPV infection, viral oncoproteins such as E6 and E7 profoundly reshape host cellular networks, targeting key regulators of DNA damage repair, cell cycle control, and innate immune surveillance. By degrading tumor suppressors like p53 and inactivating Rb, HPV promotes uncontrolled proliferation while evading immune detection. These disruptions not only facilitate viral genome replication and persistence but also create a cellular environment conducive to malignant transformation. Understanding how HPV manipulates these pathways has revealed several therapeutic opportunities—including strategies aimed at restoring p53 and Rb function, inhibiting E6/E7 activity, or enhancing innate immune responses to clear infected cells. Such targeted interventions hold promise for preventing progression from persistent HPV infection to high-grade lesions and cancer [54,55,56].

The present study demonstrates that the engineered CMV-driven vectors pUF3L1h and pUF3L2h enable robust expression of HPV L1 and HPV L2 proteins in HEK 293-T and 293-F cells, supporting the assembly of virus-like particles (VLPs). These findings expand the toolkit for dissecting HPV biology and highlight the utility of mammalian systems for producing structurally authentic capsid proteins. Importantly, the co-expression of L1 and L2 enhanced VLP stability, consistent with the established role of L2 in capsid maturation and genome encapsidation [16,17,18,20].

From a translational perspective, these vectors provide a platform for exploring novel HPV vaccine strategies. Current prophylactic vaccines rely primarily on L1-based VLPs, which elicit strong neutralizing antibody responses but are type-restricted. Incorporation of L2, which contains conserved epitopes across HPV types, may broaden protective immunity and improve cross-type coverage. The ability to co-express L1 and L2 in mammalian cells offers a valuable system for evaluating such next-generation vaccine candidates [16,17,18,20,34].

Furthermore, the modular design of pUF3/L1h and pUF3/L2h allows for the insertion of heterologous sequences, enabling studies of immune modulation, epitope presentation, and therapeutic protein delivery. By integrating tags for purification and detection, these constructs facilitate downstream biochemical and immunological analyses. Collectively, our results underscore the potential of CMV-driven L1/L2 expression vectors not only for basic virology research but also for translational applications aimed at enhancing HPV vaccine efficacy and antiviral immunity [34,37].

The HPV L1 capsid protein, regardless of the presence or absence of L2, has the property of forming structures that mimic morphologically authentic virions, VLPs, which are being used as replacements for HPV-infected cell culture in vitro. However, the presence of L2 seems to confer greater stability to the virus and VLPs; therefore, the viral capsid indeed seems to contribute to virus infectivity [37].

Most experimental models explore the process of host cell interaction by molecular or biochemical methods, using cell lines from different tissues and species in interactions with HPV L1 VLPs in their studies. In the present work, we produced HPV16 L1/L2 chimeric VLPs and applied them to investigate the possibility of these particles being internalized by human leukocytes [16,20]. We adopted a method for the isolation of these cells used in suspension at 37 °C, thereby creating an ex vivo system as closely as possible to the natural conditions of the human body [57]. HPV16 recombinant proteins L1, L2 and VLPs were visualized in the perinuclear region of HEK 293-T cells, in compliance with data described in the literature [18].

The presence of BPV (bovine papillomavirus) in lymphocytes has been discussed [58,59]. Using the PCR technique (Polymerase Chain Reaction) in samples taken from humans with anogenital lesions, the presence of HPV16 in peripheral blood samples [60] and in plasma cells was detected [61]. Additionally, DNA from other HPV types has been found in PBMC [62,63]. The presence of HPV in the lymphocytes of patients treated for anogenital cancers suggests that the virus can remain in these cells even after treatment. However, there is no convincing evidence supporting that lymphocytes carry/produce infectious HPV viral particles. Nonetheless, the presence of HPV macromolecules in lymphocytes may play a role in viral immune response. The scientific literature discloses that papillomaviruses exhibit specific tropism for species and tissues. However, BPV1 particles isolated from bovine warts and HPV16 VLPs interacted successfully with 14 cell lines derived from different species and tissues, as demonstrated in two different studies [12,13].

Using our model, we investigated the possibility of HPV16 L1/L2 chimeric VLPs crosstalk in vitro and ex vivo with two very distinct human cells, epithelial kidney cells and PBMC. After 4 h of interaction, internalization of these particles occurred, showing a very similar distribution pattern in both cell types. In both cases, L1 VLPs were in the cytoplasm near the nucleus, suggesting that tissue differences do not affect the ability of HPV16 VLPs to enter different cell types, and that both pentameric and monomeric forms of L1 could be internalized. The interaction between VLPs and monocytes observed in this study showed colocalization with CD14. As macrophage precursors, these cells can apparently internalize particles in much larger amounts compared with lymphocytes. The unexpected colocalization of the CD14 membrane receptor and VLPs, as well as the morphological changes observed, suggests that monocytes respond to this interaction as immune cells, to eliminate invading microorganisms. CD14 is a 53 kDa molecule expressed mainly in monocytes, macrophages and granulocytes. It is also found as soluble protein in the serum [64].

Viruses infect more effectively when they cluster, entering cells in groups instead of alone. These interactions require elevated cellular multiplicity of infection (MOI), which may arise through the spatial clustering of free virions or transmission modes characterized by non-independent diffusion. For viruses to infect efficiently, many particles must reach the same cell—a condition called high MOI. This can happen when viruses cluster together or spread in groups rather than individually [65].

We also investigated crosstalk between HPV16 L1/L2 chimeric VLPs and peripheral blood leukocytes via the CD71 transferrin receptor (TfR). Tf is a beta globulin responsible for the transport of iron (Fe) through plasma to the inside of cells. Besides hepatocytes, other cells in the body can synthesize it. The TfR is a membrane protein expressed primarily in T and B lymphocytes, macrophages, and proliferating cells such as homodimers with N-terminal cytoplasmic tails [66]. TfR is constantly being endocytosed by the plasma membrane through clathrin-mediated endocytosis, with the purpose of carrying ferric iron bound to Tf into the cells. This Tf-TfR complex is delivered to the early endosome into a lower pH medium, where iron is released and TfR is recycled to the cell surface [67]. When evaluating the interactions between HPV16 L1/L2 chimeric VLPs, HPV6, 11, 16, 18 L1 VLPs (4-valent Gardasil^®^ vaccine, MSD) and unstructured HPV16 L1 in PBMCs in the presence of exogenous Tf, we determined the colocalization of L1-VLP-Tf and free Tf in these cells, as well as colocalization with TfR, after 45 min of contact. This colocalization between VLP, Tf and TfR was found in approximately 50% of analyzed cells. These results suggest that HPV16 L1/L2 chimeric VLPs, HPV6, 11, 16, 18 L1 VLPs (Gardasil^®^ vaccine) and unstructured HPV16 L1 can enter cells by endocytosis through clathrin dependence, via TfR. We hypothesized that during viral infection processes, interactions between HPV16 (about 60 nm in size) and Tf molecules (80 kDa) might indeed happen through fusion with Fe-Tf-TfR complexes, mimicking their entry into the cell via low pH endosomes, thus enabling transcytosis [51,52,67,68].

There have been no reports in the literature showing the crosstalk of HPV VLPs in human PBMCs. Up to the point in time under discussion, this study has shown that PBMCs of healthy female volunteers were able to internalize HPV16 L1/L2 chimeric VLPs, as well as HPV6,11,16,18 L1 chimeric VLPs (4-valent Gardasil^®^ vaccine, MSD). In addition, T and B lymphocytes were the main cell types that further internalized VLPs. It is important to emphasize that lymphocytes are cells capable of dividing themselves, and it is estimated that the half-life of these inactive cells in humans is several years. Furthermore, inactive lymphocytes circulate continuously through the bloodstream and lymphatic vessels. The entry of HPV VLPs into leukocytes by independent endocytosis pathways may be due to their ability to recognize, capture and remove foreign substances from the body. The use of different biochemical inhibitors, whether isolated or in combination, does not seem to be enough to block the uptake of HPV16 L1/L2 chimeric VLPs in PBMCs. However, to understand the possible mechanisms involved in these PBMC interactions in future studies, which in turn cannot be assessed only by VLP experiments, it will be necessary to develop new strategies using PsV (pseudovirion) infectious particles provided with the HPV16 viral genome [55,69,70].

Thinking from another approach, it will be possible to exploit this experimental model in vaccine–cell interaction studies and the immunological mechanisms involved, for instance, by evaluating the impact of vaccines and the prevalence of specific viral genotypes in distinct populations [71,72,73,74]. Interestingly, a review discusses the importance of clarifying some lapses in our understanding of HPV natural history [75]. A study demonstrates the transmission of papillomavirus through blood and the induction of infection in mice and rabbits that received this contaminated blood [76].

Recent research evaluated HPV prevalence, genotype distribution, and associations with cervicovaginal microbiota and cytokine profiles, based on a systematic review and meta-analysis among South African women, where cervical cancer ranks as the second most common cancer. Cytokine analysis presented elevated MIP-1α and MIP-1β in HPV infections, though cytokine profiles may depend on HPV genotypes. These findings point to the need for research on HPV-microbiome-immune interactions and call for comprehensive HPV-prevention strategies, including vaccines targeting regional HPV types and tailored interventions for HIV-positive populations [77].

Carcinogenic HPVs are directly related to the induction of cervical cancer. HPV vaccines are satisfactorily immunogenic in people living with HIV, but further evaluation is needed on the long-term impact of vaccination and to determine whether more boosters are required [78]. There are six licensed prophylactic vaccines, but there is still no approved therapeutic vaccine against HPV globally. There are only those in pre-clinical and clinical trials [37,43].

Some HPV specialists have discredited the possibility of HPV remaining in a latent state in the body, which would justify its manifestation at times other than during infection, for example, when there is a decline in immunity in an asymptomatic HPV carrier for a long time. A working group formed by the International Papillomavirus Society to consider the latency of cervical HPV has led to an updated understanding of the natural history of HPV [79]. The previous model considered HPV detection as resulting from acquisition or potential reinfection by the virus, and loss of detection as resulting from viral elimination; the updated understanding of the natural history of HPV is more complex. It is now considered that HPV detection can occur as a result of autoinoculation, deposition from a recent sexual act, or as a recurrence of a previously acquired infection [79]. The working group also considers that loss of HPV detection likely reflects immune control rather than complete viral elimination. Since it is virtually impossible to identify the exact source of a new HPV detection or to determine why HPV is no longer detectable, it is recommended that healthcare professionals and researchers use the terminology “HPV detected” versus “HPV not detected” [79]. Furthermore, they describe the updated understanding in a clinical context. Specifically, they discuss the potential implications of this updated understanding regarding clinical counseling on screening, recommendations on cervical cancer screening, and human papillomavirus vaccination. They also suggest key phrases that healthcare professionals can use when counseling women attending routine human papillomavirus-based cervical cancer screening [79].

Cervical samples remain the gold standard for HPV detection owing to their superior diagnostic accuracy. Self-collected cervicovaginal specimens have been validated as reliable alternatives and are particularly valuable for improving participation rates in screening programs. Urine samples, especially first-void collections, show strong potential for large-scale screening and vaccine impact monitoring, with meta-analyses confirming sensitivity comparable to clinician-collected specimens. Blood-based assays hold promise for detecting circulating HPV DNA and monitoring treatment response in HPV-driven cancers, though they are not yet suitable for routine screening. Oral samples are most relevant in the context of oropharyngeal cancer research; however, their moderate sensitivity currently limits their application in secondary prevention strategies [25].

Urine and self-collected specimens require rigorous standardization to ensure reproducibility and methodological consistency. Importantly, alternative sampling approaches may broaden population coverage, extending screening access to underserved groups. Nevertheless, diagnostic accuracy remains variable across specimen types, with cervical samples continuing to represent the benchmark for sensitivity and specificity in primary screening. At present, blood and oral specimens are more appropriately applied within research and prognostic monitoring contexts, rather than routine clinical screening programs [25].

From this new perspective, the experimental results reported in this article support the hypothesis of HPV latency in previously infected individuals who later experience recurrence, transmit the virus, and eventually develop HPV-positive cancers. Furthermore, our findings validate HPV16 L1/L2 chimeric VLPs as efficient therapeutic immunogens, with promising potential not only as prophylactic nanovaccines but also as therapeutic drug nanocarriers for additional applications [37].

## 4. Materials and Methods

### 4.1. Production of HPV16 L1/L2 Chimeric VLPs

HEK 293 cells, originating in 1973, possess multiple variants derived from the same lineage, used for the production of recombinant proteins, virus-like particles (VLPs), and viral vectors. The introduction of adenoviral genes promotes their immortalization, supporting cell growth and adeno-associated virus (AAV) production, as well as facilitating transfection and generating specific lineages. HEK 293 variants have been selected for specific functional properties, aiming to optimize yield, quality, stability, and adaptation to large-scale production environments [16,17,18,20]. Despite their traditional use in research, there is growing interest in their industrial application, especially in the production of viral vectors and therapeutics, requiring a greater understanding of fundamental biological factors for optimization [80,81].

A systemic understanding of HEK 293 cells, including genomic, transcriptomic, proteomic, metabolomic, and lipid analyses, has allowed the identification of differences between variants, supported line engineering, and improved productivity. Engineering efforts include promoter modification, identification of genomic “safe harbors” for gene insertion, and development of gene selection and amplification systems, such as those deficient in DHFR (Dihydrofolate reductase) or GS (Glutamine synthase), to increase the expression of recombinant proteins [80,82].

HEK 293 cells represent robust and reliable platforms with the potential for expansion in the production of therapeutics, viral vectors, and vaccines, especially with advances in adaptation to large-scale culture processes and molecular characterization. The integration of knowledge from fundamental biology, genetic engineering, and bioprocesses will allow the development of more efficient, safe, and scalable cell lines. The exchange of experiences between protein production communities, VLPs, and viral vectors, in addition to the use of common technologies, will facilitate innovation and the implementation of more advanced production platforms. In brief, HEK 293 cells, with their engineering and adaptation potential, are positioned to consolidate themselves as central platforms in modern biotechnology, especially in the production of viral therapeutics and vaccines, driven by advances in molecular characterization and manufacturing processes [80].

The production of VLPs in this research was carried out throughout the recombinant protein expression of HPV16 L1 and L2 in epithelial human cells of the HEK 293-T and HEK 293-F cell lineages, cultured as previously described in detail [16,20]. Transfection of these cells occurs in a transient manner using pUF3/L1h and pUF3/L2h vectors (Figure 1, Figure 2 and Figure 23), which are regulated by the human cytomegalovirus promoter containing complete sequences of the L1 and L2 genes [14]. In this study, besides being used to produce VLPs containing both capsid proteins, the HEK 293-T cell line was also used as a control factor in crosstalk assays with human peripheral blood leukocytes (Figure 8, Figure 11A and Figure 12). Conversely, HEK 293-F produced VLPs for crosstalk leukocyte assays (Figure 11B’).

A potential limitation of employing human cell lines is the risk of human-specific viral contamination, though this can be effectively mitigated through multiple viral inactivation and clearance procedures. Although human cell lines are increasingly utilized in biopharmaceutical research, vaccine development, and the manufacture of certain licensed protein therapeutics, clinical experience with them remains relatively limited. This is largely because their application in protein production has only recently begun, in contrast to other, more established cell line platforms. With sustained research investment, human cell lines could be further optimized to enable routine commercial production of a broader spectrum of biotherapeutic proteins [82,83,84,85].

### 4.2. Cell Culture/Cell Line

Freestyle™ 293-F (Invitrogen™, Carlsbad, CA, USA): A derivative of the HEK 293 cell line, adapted for suspension growth (Invitrogen Manual, 2007).

HEK 293 cells originate from human embryonic kidney cells transformed with human adenovirus type 5 (Ad5) DNA [86].

#### Suspension Cell Culture

Freestyle™ 293 Expression System (Invitrogen™, Carlsbad, CA, USA): The 293-expression system includes the serum-free FreeStyle™ 293 Expression Medium.

FreeStyle™ 293 F (293 F) cells were cultured in suspension at a density of 3 × 10^5^ viable cells/mL in serum-free 293 Expression Medium, following the manufacturer’s recommendations. Cultures were subpassaged when cell density reached 2–3 × 10^6^ viable cells/mL, typically every 3–4 days. Aliquots of the suspension were transferred and vigorously homogenized for 10–30 s to disrupt cell aggregates. Cell concentration and viability were determined by trypan blue exclusion using a Neubauer chamber and visualized with a Leica DMIL inverted microscope. Suspension cultures were maintained at 37 °C in a humidified atmosphere containing 5% CO_2_, under orbital agitation at 120 rpm. For cell banking, suspensions were cryopreserved in cryovials at a density of 5–8 × 10^6^ cells/mL in freezing medium consisting of 90% serum-free 293 Expression Medium and 10% dimethyl sulfoxide (DMSO), and stored in liquid nitrogen at −192 °C [20].

The humanized gene expression vectors pUF3/L1h and pUF3/L2h (Figure 1 and Figure 2), used in cloning, transfection, and co-transfection experiments, were kindly provided by Prof. Dr. Martin Müller (Forschungsschwerpunkt Angewandte Tumorvirologie, DKFZ, Heidelberg, Germany) [14].

The vectors were constructed under the control of the constitutive human cytomegalovirus (pCMV) promoter, driving the expression of the HPV16 L1 and L2 genes, respectively. Both vectors contain the ampicillin resistance gene and the reporter gene encoding green fluorescent protein (GFP). We have substituted ampicillin with kanamycin in both genetic constructions [40].

### 4.3. Plasmid Vector Amplification

#### 4.3.1. Preparation of Competent Bacteria

*Escherichia coli* DH5α, a strain suitable for genetic manipulations and widely used for plasmid DNA propagation and gene cloning, was employed. Competent cells were prepared following the method described by [87]. *E. coli* DH5α cells were cultured in Luria-Broth (LB) medium (1% tryptone, 0.5% yeast extract, 0.5% NaCl) at 37 °C with agitation at 250 rpm until reaching an OD_600_ of 0.6. Cells were harvested by centrifugation at 5000× *g* for 10 min at 4 °C, resuspended in ice-cold 0.1 M CaCl_2_, and incubated on ice for 60 min. After a second centrifugation under the same conditions, the pellet was resuspended in 2 mL of ice-cold 0.1 M CaCl_2_. Competent cells were aliquoted in 10% glycerol and stored at −80 °C until use.

#### 4.3.2. Transformation of Competent Bacteria

Transformation was performed according to [88]. Briefly, 10 μL of plasmid DNA was added to chemically competent E. coli DH5α cells. The mixture was incubated on ice for 30 min, subjected to heat shock at 42 °C for 2 min, and returned to ice for 5 min. Subsequently, 350 μL of LB medium was added, and the suspension incubated at 37 °C for 90 min. The transformed cells were plated on LB agar containing 100 μg/mL ampicillin and incubated at 37 °C for 18 h.

#### 4.3.3. Selection of Recombinant Clones and Plasmid DNA Extraction

Ampicillin-resistant E. coli DH5α colonies were selected and inoculated into 3 mL of LB medium supplemented with 100 μg/mL ampicillin. Cultures were incubated at 37 °C for 18 h with agitation at 250 rpm. Cells were harvested by centrifugation at 12,000× *g* for 30 s, and plasmid DNA was purified using the AxyPrep™ Plasmid Miniprep Kit (Axygen Biosciences, Union City, CA, USA), following the manufacturer’s instructions. DNA concentration was determined using a Nanodrop™ 100 spectrophotometer (ThermoScientific, Waltham, MA, USA). Purified plasmid DNA aliquots were stored at −20 °C until further use.

### 4.4. Cell Transfection

Transient transfections and co-transfections in FreeStyle™ 293-F cells were performed by lipofection using the 293fectin™ reagent (Invitrogen™), according to the manufacturer’s instructions. The cells were seeded one day prior to transfection at a density of 6.0–7.0 × 10^5^ viable cells/mL in serum-free 293 Expression Medium. On the day of transfection, 3 × 10^7^ cells were maintained in 28 mL of medium at 37 °C in a humidified atmosphere with 5% CO_2_ under orbital agitation at 120 rpm. For single transfections, 30 μg of L1 plasmid DNA was used; for co-transfections, 15 μg of L1 and 15 μg of L2 plasmid DNA were employed. DNA was diluted in 1 mL of Opti-MEM^®^ I (Invitrogen™), while 60 μL of 293fectin™ reagent was diluted in 1 mL of Opti-MEM^®^ I, gently mixed, and incubated at room temperature for 5 min. The two solutions were combined, gently homogenized, and incubated for 30 min at room temperature. The resulting 2 mL transfection mixture was added to the cell suspension. Transfected and co-transfected 293-F cells were collected at various time points (6–72 h post-transfection) for kinetic analysis of HPV16 L1 and L2 recombinant protein expression. Flow cytometry, confocal laser microscopy, epifluorescence microscopy, and transmission electron microscopy (TEM) were employed to evaluate protein expression.

### 4.5. Expression of Recombinant L1 and L2 Proteins

#### 4.5.1. Confocal and Epifluorescence Microscopy

For the detection of HPV16 L1 and L2 recombinant proteins, 293-F cells were cultured and transfected as previously described. The cells were washed three times with PBS (pH 7.4) and centrifuged at 200 ×g for 5 min. They were incubated in 1 mL PBS containing 5% bovine serum albumin (BSA, Sigma-Aldrich^®^, St. Louis, MO, USA) for 60 min, followed by three washes. After centrifugation, the cells were incubated with primary antibodies: monoclonal anti-HPV16 L1 (Camvir-1, BD Pharmingen^®^, San Diego, CA, USA) and/or polyclonal anti-HPV16 L2 (11–200 aa), kindly provided by Dr. Richard Roden (Department of Pathology, Johns Hopkins University, Baltimore, MD, USA). Antibodies were diluted 1:150 and 1:100, respectively, in PBS containing 0.01% Tween 20 and 0.5% BSA, and incubated for 2 h at room temperature. The cells were washed with PBS containing 1% BSA and incubated with secondary antibodies: goat anti-mouse IgG conjugated to AlexaFluor^®^ 488 (for L1 detection) and goat anti-rabbit IgG conjugated to AlexaFluor^®^ 633 (for L2 detection) (Molecular Probes™, Carlsbad, CA, USA). Both were diluted 1:250 in PBS containing 0.01% Tween 20 and 1.5% BSA, and incubated for 1 h at room temperature. After washing, the cells were mounted on poly-L-lysine–treated slides (Sigma-Aldrich^®^) with 5 μL Mowiol (EMD Biosciences, La Jolla, CA, USA) and stored at 4 °C protected from light until analysis using a Zeiss LSM Meta confocal laser scanning microscope (Zeiss, Germany) at the Parasitology Laboratory, Butantan Institute. For nucleic acid staining, fixed cells were mounted with 10 μL Vectashield^®^ containing DAPI (Vector Laboratories, Burlingame, CA, USA) and analyzed using a Nikon Eclipse Ni epifluorescence microscope (Nikon Corporation, Shinagawa-ku, Tokyo, Japan) at the Genetics Laboratory, Butantan Institute.

#### 4.5.2. Flow Cytometry

For detection of HPV16 L1 and L2 proteins in 293-F cells by flow cytometry, cells were cultured and transfected as described above. The transfected cells were washed three times with PBS (pH 7.4) and centrifuged at 200× *g* for 5 min. The cells were incubated in 1 mL PBS (pH 7.4) containing 3% bovine serum albumin (BSA) and 0.05% Tween 20 for 60 min, followed by washing in PBS. After centrifugation, the cells were incubated with primary antibodies anti-HPV16 L1 and/or anti-HPV16 L2, diluted 1:150 and 1:100, respectively, in PBS containing 0.01% Tween 20 and 0.5% BSA for 60 min at room temperature. The cells were then washed and incubated with secondary antibodies: goat anti-mouse IgG conjugated to AlexaFluor^®^ 488 and goat anti-rabbit IgG conjugated to AlexaFluor^®^ 546 (Molecular Probes™, Carlsbad, CA, USA), both diluted 1:250 in PBS containing 3% BSA for 60 min at room temperature. After final washes, the cells were analyzed using a BD FACSCanto™ II flow cytometer with BD FACS Diva software (BD Biosciences, San Jose, CA, USA) at the Butantan Institute.

#### 4.5.3. Transmission Electron Microscopy (TEM)

Non-transfected 293-F cells were cultured to assess normal morphology (Figure 6) and serve as negative controls for L1 and L2 protein expression, as previously described [16,18]. The cells were cultured under the conditions previously outlined, washed with PBS, and centrifuged at 200× *g* for 5 min. The pellet was washed three times with PBS to remove culture medium residues and cellular debris, then fixed in 0.1 M sodium phosphate buffer (pH 7.2) containing 3.5% sucrose and 2.5% glutaraldehyde (Merck, Darmstadt, Germany) for 1 h at room temperature. The cells were washed three times in the same buffer and centrifuged, followed by post-fixation in 1% osmium tetroxide (Sigma-Aldrich^®^, St. Louis, MO, USA) for 1 h at room temperature, protected from light with occasional agitation. Samples were washed in phosphate buffer containing sucrose, then twice in 0.85% saline and once in distilled water (10 min each).

For nucleic acid contrast, the cells were incubated with 2% aqueous uranyl acetate for 2 h at room temperature, washed three times in double-distilled water, and dehydrated through a graded ethanol series (30%, 50%, 70%, and 95% for 15 min each; 100% ethanol for 30 min), followed by ethanol–acetone (1:1) and 100% acetone. The cells were infiltrated with Epon resin (Polybed Kit, Polysciences Inc., Warrington, PA, USA) using acetone–Epon mixtures (2:1 and 1:1 for 30 min each, then 1:2 overnight at 4 °C). After overnight incubation, the samples were embedded in pure Epon resin for 24 h, centrifuged at 1250× *g* for 30 min, and replaced with fresh resin for 4 h at room temperature. The cells were transferred to gelatin capsules filled with fresh resin and polymerized at 60 °C for 72 h. Ultrathin sections were prepared using an ultramicrotome, mounted on copper grids (300 mesh) pre-coated with 2% parlodion in amyl acetate and carbon, and contrasted with 2% aqueous uranyl acetate (Polysciences Inc.). After drying at room temperature, samples were examined with a Zeiss EM 109 TEM operated at 80 kV at the Genetics Laboratory, Butantan Institute. Images were acquired using a Mega View III camera and iTEM Basic Version software (Olympus Soft Imaging Solutions GmbH, Münster, Germany).

### 4.6. HPV16 L1 and L2 Proteins Ultrastructural Immunocytochemistry

#### 4.6.1. Analysis of Virus-like Particles (VLPs)

For immunocytochemical assays, 10 μL of clarified cell lysate samples were applied to nickel grids (300 mesh) coated with 2% collodion in amyl acetate and carbon for 1 min at room temperature. Excess sample was removed with filter paper. Grids were incubated with primary antibodies anti-HPV16 L1 and anti-HPV16 L2, diluted 1:100 in PBS containing 0.01% Tween 20 and 0.5% BSA, for 2 h in a humid chamber at room temperature. After washing in PBS, grids were incubated with secondary antibodies: goat anti-mouse IgG and goat anti-rabbit IgG (Sigma-Aldrich^®^), conjugated to colloidal gold particles (10 nm for L1, 15 nm for L2), diluted 1:100 in PBS containing 0.01% Tween 20 and 1.5% BSA, for 1 h at room temperature. Grids were washed four times in saline containing 1% BSA (5 min each) and once with distilled water using gentle jets to remove nonspecific particles. Negative staining was performed with 2% aqueous uranyl acetate for 1 min. After drying at room temperature, samples were examined with a Zeiss EM 109 TEM operated at 80 kV at the Genetics Laboratory, Butantan Institute. Images were acquired using a Mega View III camera and iTEM Basic Version software (Olympus Soft Imaging Solutions GmbH, Münster, Germany).

#### 4.6.2. Preparation of Clarified Cell Lysates

HEK 293-F cells were cultured and transfected as previously described. Non-transfected and co-transfected cells (L1 and L2 vectors) were harvested 48 h post-transfection, washed three times in PBS (pH 7.4), and centrifuged at 200× *g* for 10 min. The resulting pellet was resuspended in lysis buffer containing protease inhibitors (Sigma Fast™ Protease Inhibitor Tablets, Sigma-Aldrich^®^, St. Louis, MO, USA) according to the manufacturer’s instructions. The cells were lysed by five cycles of vortex agitation followed by 1 min incubation on ice. Lysates were clarified by centrifugation at 500× *g* for 10 min, and supernatants were stored at −20 °C until analysis.

### 4.7. Purification of HPV16 L1 and L2 Proteins

#### 4.7.1. Ammonium Sulfate Precipitation

For the purification of intracellular HPV16 L1/L2 proteins, co-transfected cells were lysed as described above. Clarified lysates were precipitated with 45% ammonium sulfate, homogenized at 4 °C for 30 min, and centrifuged at 12,000 ×g for 10 min at 4 °C. The supernatant was discarded, and the pellet resuspended in 1 mL sterile PBS (pH 7.2). The samples were dialyzed against a phosphate buffer (pH 7.2) containing 0.01% Tween 80 at 4 °C for 72 h, with buffer changes every 12 h.

#### 4.7.2. Size-Exclusion Chromatography

Following dialysis, samples were subjected to size-exclusion chromatography using Sephacryl™ S-300 HR resin (Sigma-Aldrich^®^, St. Louis, MO, USA) packed into a 10 mL polypropylene column (Sigma-Aldrich^®^) connected to a peristaltic pump (PUMP P-50, Amersham Biosciences). The column was manually packed with 3 mL of resin, washed with distilled water to remove preservative, and equilibrated with 5 column volumes of buffer (10 mM sodium phosphate, pH 7.2; 0.15 M NaCl; 0.01% Tween 80). A 0.5 mL aliquot of clarified lysate, with total protein content determined by the bicinchoninic acid (BCA) assay, was applied at a flow rate of 0.5 mL/min. Proteins were eluted with the same buffer, and fractions of 0.25 mL were collected in siliconized microtubes. After chromatography, the column was washed with distilled water and stored in 20% ethanol at 4 °C. Purified fractions were analyzed for protein concentration, SDS-PAGE, and Western blotting.

#### 4.7.3. Affinity Chromatography

An alternative purification strategy for HPV16 L1/L2 proteins was performed using affinity chromatography. Protein precipitation with 45% ammonium sulfate and dialysis were carried out as previously described. Purification was performed with a pre-packed HiTrap™ Heparin HP column (GE Healthcare, Uppsala, Sweden) connected to a peristaltic pump (PUMP P-50, Amersham Biosciences). The column was washed with 5 column volumes (CV) of distilled water to remove the preservative solution, then equilibrated with 5 CV of sodium phosphate buffer (pH 7.0) containing 0.3 M NaCl and 0.01% Tween 80. A 0.3 mL sample was applied at a flow rate of 0.5 mL/min, followed by washing with sodium phosphate buffer (pH 7.2) containing 0.3 M NaCl and 0.01% Tween 80. Bound proteins were eluted with sodium phosphate buffer (pH 7.2) containing 1.0 M NaCl and 0.01% Tween 80 at a constant flow rate of 0.5 mL/min. Fractions of 0.25 mL were collected in siliconized microtubes. The column was subsequently washed with distilled water, stored in 20% ethanol at 4 °C, and reused as needed. Eluted fractions were analyzed for protein concentration, SDS-PAGE, and Western blotting.

#### 4.7.4. Protein Quantification

Protein concentrations during purification and characterization assays were determined using the BCA Protein Assay Kit (Pierce, Rockford, IL, USA), based on the bicinchoninic acid method. Microassays were performed according to the manufacturer’s instructions, detecting protein concentrations between 5 and 250 μg/mL. In a 96-well microplate, 25 μL of test samples or BSA standards were pipetted into each well. Subsequently, 200 μL of reagent mixture (50 parts reagent A to 1 part reagent B) was added. Plates were incubated at 37 °C for 30 min, and absorbance was measured at 570 nm using a Multiskan Ex plate reader (Labsystems Uniscience, Sao Paulo, Brazil) at the Genetics Laboratory, Butantan Institute. Protein content was calculated using BSA as the standard.

### 4.8. Characterization of L1 and L2 Proteins

#### 4.8.1. SDS-PAGE (10%)

Purified fractions, clarified L1/L2 cell lysates, and non-transfected 293-F cells were analyzed for purity by sodium dodecyl sulfate–polyacrylamide gel electrophoresis (SDS-PAGE) [89]. Separation gels contained 10% polyacrylamide. Samples were diluted in sample buffer containing 62.5 mM Tris/HCl (pH 6.8), 10% SDS, 0.02% bromophenol blue, 20% glycerol, and 2% β-mercaptoethanol under reducing conditions. BenchMark™ Protein Ladder (Invitrogen™, Carlsbad, CA, USA) was used as the molecular weight standard. After electrophoresis, gels were stained using the silver staining method [90].

#### 4.8.2. Western Blotting

The samples were subjected to SDS-PAGE (10%) under reducing conditions, as described above. Proteins were transferred to nitrocellulose membranes (Trans-Blot Transfer Membrane, Bio-Rad, Benicia, CA, USA) using a Trans-Blot SD transfer system (Bio-Rad) with transfer buffer containing 250 mM glycine, 1% SDS, and 20% methanol in 25 mM Tris-HCl (pH 8.3), at 400 mA for 1 h at 4 °C. Membranes were stained with Ponceau solution to verify transfer efficiency and identify molecular weight marker bands (BenchMark™ Protein Ladder, Invitrogen™).

The membranes were blocked overnight at 4 °C in PBS-T (0.05% Tween 20) containing 5% skim milk, then washed three times in PBS-T (10 min each). Primary antibody incubation was performed for 1 h at room temperature with gentle agitation: monoclonal anti-HPV16 L1 (Camvir-1, 1:500 dilution) and polyclonal anti-HPV16 L2 (1:50 dilution) or monoclonal anti-HPV16 L2 (1–40 aa, Santa Cruz Biotechnology, Dallas, TX, USA; 1:10 dilution). After washing, membranes were incubated with HRP-conjugated secondary antibodies (goat anti-mouse IgG for L1 and goat anti-rabbit IgG for L2, both diluted 1:500 in PBS-T with skim milk) for 1 h at room temperature. Following three washes, detection was performed using the Novex^®^ ECL HRP Chemiluminescent Substrate Reagent Kit (Invitrogen™). Membranes were exposed to Amersham Hyperfilm™ ECL (GE Healthcare, Hatfield, UK) in a darkroom. Films were developed and fixed using Kodak reagents, air-dried, and digitized with an HP Scanjet G4050 scanner and HP Photosmart Premier Version 7.0 software.

### 4.9. Immunogenicity of L1/L2 VLPs

#### 4.9.1. Animal Immunization

To evaluate the immune response induced by L1/L2 VLPs, groups of six female Balb/c mice (6–8 weeks old) were obtained from the Central Animal Facility of the Butantan Institute, with approval from the Ethical Committee. Vaccine formulations were prepared with 20 μg of affinity-purified L1/L2 proteins, dialyzed in buffer (pH 7.4) containing 0.3 M NaCl and 0.01% Tween 80. Dialyzed samples were diluted in 250 μL of buffers containing 200 μg of aluminum hydroxide (Al (OH)_3_) as an adjuvant. The mice were immunized subcutaneously with three doses of 250 μL vaccine formulations at 15-day intervals (days 0, 15, and 30).

One group received VLPs formulated with aluminum hydroxide, a second group received 20 μg of VLPs without adjuvant, and a control group received 0.85% saline solution. Two weeks after the third immunization, blood samples were collected for antibody analysis. Pre-immune sera were collected prior to the first immunization. After collection, sera were incubated at 37 °C for 30 min, held at 8 °C for 30 min, centrifuged, and stored at −20 °C until use in subsequent assays.

#### 4.9.2. Titration of Anti-HPV16 L1 and L2 Sera

Serological titration of anti-HPV16 L1 and L2 antibodies was performed using an indirect enzyme-linked immunosorbent assay (ELISA). Flat-bottom 96-well microplates were coated with HPV16 L1 or L2 proteins (various concentrations) diluted in 100 μL of carbonate buffer (100 mM, pH 9.6) and incubated overnight at 4 °C in a humid chamber. Wells were washed three times with PBS containing 0.05% Tween 20 (PBS-T) and blocked with 200 μL of 3% BSA in PBS for 2 h at 37 °C. After washing, serial dilutions (starting at 1:10 or 1:50) of control sera and pooled sera from immunized animals (with or without adjuvant) were prepared in PBS containing 1% BSA and added to the wells (100 μL per well). Plates were incubated for 2 h at 37 °C.

After washing, 100 μL of HRP-conjugated goat anti-mouse IgG (Sigma-Aldrich^®^), diluted 1:3000 in PBS-T, was added and incubated for 1 h at 37 °C. The plates were washed and developed with 100 μL of citrate–phosphate buffer (pH 5.0) containing ortho-phenylenediamine (OPD) and hydrogen peroxide (H_2_O_2_) for 15 min at room temperature in the dark. The reaction was stopped with 50 μL of 4.5 N sulfuric acid (H_2_SO_4_), and absorbance was measured at 492 nm using a Multiskan Ex plate reader (Labsystems Uniscience) [91,92,93].

#### 4.9.3. Antibody Characterization

The specificity and reactivity of sera from immunized mice were assessed by Western blotting and ELISA, as already described. Neutralization assays were performed to evaluate the functional activity of antibodies against HPV16 L1/L2 virus-like particles (VLPs). Briefly, sera were incubated with HPV16 pseudovirions under standardized conditions, and infectivity was measured in susceptible cell cultures. Reduction in infection relative to control sera was used to determine neutralizing antibody titers.

#### 4.9.4. Statistical Analysis

All experimental data were expressed as mean ± standard deviation (SD). Statistical significance was determined using one-way analysis of variance (ANOVA) followed by Tukey’s post hoc test, with *p* < 0.05 considered significant. GraphPad Prism Version 8.0 software (GraphPad Software Inc., San Diego, CA, USA) was used for data analysis and graph generation.

### 4.10. Blood Collection

To obtain the samples, we relied on the collaboration of Prof. Dr. Elizabeth Leão, MD, São Joaquim Hospital, of the Royal and Meritorious Portuguese Beneficent Association, SP, Brazil, to ensure the uniformity of the information. Blood samples were requested from 10 healthy female volunteers, aged between 35 and 55 years, based on epidemiological data associated with genital HPV infection. The screening of volunteers was carried out based on recent blood counts, Pap smears and colposcopy, and on data provided by the candidates, through the completion of a free and informed consent form (Annex A) and questionnaire (Annex B) for the purpose of laboratory research—“Questionnaire for collecting information on sexual behavior and other possible factors associated with HPV infection and anogenital cancer”. Volunteers who did not present blood count, Pap smear, and colposcopy with normal reference values, and those who provided information on sexual behavior and other important epidemiological factors correlated with HPV infection that could compromise the research results, were dismissed. Blood samples were collected in sterile tubes containing heparin, for internalization assays with leukocytes, and EDTA for blocking assays, and were processed quickly, within a maximum of 2-h after collection. During this interval, they were stored at 4 °C [57].

### 4.11. Selection of Receptors

The choice of receptors in HPV VLP–leukocyte interaction studies often depend on the experimental focus (Table 1, Table 2, Table 3 and Table 4). Researchers typically highlight receptors that are well-documented in HPV entry pathways, such as heparan sulfate proteoglycans (HSPGs) on the cell surface, because they serve as the primary attachment factors for HPV particles. After binding, conformational changes in the capsid expose epitopes that allow engagement with secondary receptors or co-receptors, leading to internalization.

Integrin α6 (often paired with β4) has indeed been implicated as a co-receptor in HPV entry, particularly in epithelial cells. However, its role is more cell-type specific. Leukocytes do not consistently express integrin α6 at the same levels as epithelial cells, so in leukocyte-focused assays, researchers may prioritize receptors that are broadly expressed and experimentally validated in that context. In other words, the receptor choice reflects both biological relevance (what the cells actually express) and methodological clarity (what can be reliably detected and interpreted in immunofluorescence assays).

So, the omission of integrin α6 is not necessarily a denial of its importance; it is more about tailoring the receptor analysis to the cell type under study. If the goal were to explore epithelial infection pathways, integrin α6 binding would be a stronger candidate to highlight.

### 4.12. Leukocyte Separation

Whole blood was collected in heparin at a concentration of 0.1 mg/mL, centrifuged at 1000 rpm for 7 min at 5 °C in a Sorvall^®^ RT 6000 Refrigerated Centrifuge (Du Pont, Wilmington, DE, USA) with a horizontal angle rotor. The plasma supernatant containing platelets and leukocytes was gently removed and transferred with the aid of a Pasteur pipette to a new tube. An aliquot was collected for total and differential counts, performed in a Neubauer chamber, diluted (ratio 1:1) in Trypan blue, and smears were stained with May–Grünwald–Giemsa stain to determine their composition. The sedimented leukocytes were centrifuged again at 1200 rpm, for 3 min, at 5 °C, to remove platelets. Leukocytes precipitates were resuspended in a 0.85% saline solution for subsequent tests [57]. The Neubauer chamber counts and smears containing distinct cell types were analyzed by light microscopy using a Leica DMIL I microscope (Leica Microsystems GmbH, Vienna, AUT).

### 4.13. Internalization Assays for HEK 293-T Cells and HPV16 L1/L2 VLPs

HEK 293-T cells (2 × 10^4^ cells/mL) were plated and maintained under growth conditions, washed with PBS and incubated with 120 µg of VLPs in DMEM without FBS for 4 h at 37 °C and 5% CO_2_. After this, the cells were rinsed twice with PBS for 3 min each to remove non-internalized particles. The cells were then fixed with 2% PFA (Paraformaldehyde, Sigma-Aldrich) in PBS for 1 h at 4 °C and rinsed three times with PBS for 5 min each. The plates were kept at 4 °C until immunofluorescence assays. Controls were performed in the absence of VLPs and/or with the denaturation thereof, by heating at 100 °C for 10 min. Adapted from [94].

### 4.14. Internalization Assays for Human Leukocytes and HPV16 L1/L2 VLPs

Leukocytes (2 × 10^4^ cells/mL) from healthy volunteers were incubated with 120 µg of VLPs produced in this study and RPMI without FBS for 4-h at 37 °C and 5% CO_2_, under gentle agitation. The cells were centrifuged at 1000 rpm for 7 min, and cell pellets were washed three times with PBS, for 5 min each. Then, cells were fixed with 2% PFA in PBS for 1 h, at 4 °C. After this step, the centrifugation and PBS washing processes were repeated. The samples were kept at 4 °C until immunofluorescence assays. Controls were the same as described above for HEK 293-T cells.

### 4.15. Internalization Assays for Leukocytes, Transferrin and HPV VLPs

The same protocol described above was employed, with minor changes described below. Leukocytes were incubated with 120 µg of VLPs in separate samples, as follows: the HPV16 L1/L2 VLPs produced in this study and L1 VLPs of HPV6, 11, 16 and 18 from the Gardasil^®^ vaccine, used as control, kindly provided by Merck Sharp & Dohme. RPMI without FBS, together with transferrin (Tf) conjugated to the fluorochrome TexasRed^®^ (Molecular Probes™), was added (ratio 1:60, Tf: RPMI). Samples were kept for 15, 45, 60 and 120 min in an incubator at 37 °C and 5% CO_2_, under gentle agitation. The other procedures remained unchanged.

### 4.16. Blocking Assays for Membrane Receptors

Leukocytes from healthy donors were counted and incubated in RPMI medium overnight on coverslips containing poly L-lysine (Sigma-Aldrich) at 37 °C and 5% CO_2_. Then, the cells were rinsed, and a fresh medium was added together with specific biochemical inhibitors of ligand uptake (Table 1), used either in isolation or in combination, and incubated for 2 h [94]. After incubation, the cells were rinsed and 120 µg of HPV16 L1/L2 VLPs [95] was added and incubated with the medium for 4 h. The cells were rinsed again and fixed in a 2% PFA solution. Immunofluorescence assays were carried out to detect the VLP-PBMC interaction by confocal microscopy, using specific antibodies to recognize L1 and L2 proteins. Z-axis 3D images were obtained to confirm the presence of VLPs within the cells.

### 4.17. Immunofluorescence Assays for Crosstalk Analysis

The leukocytes were washed and fixed as already described. The samples were incubated in PBS containing 1% BSA for 5 min under gentle agitation. Later, the cells were incubated with the primary antibodies (Table 1), diluted in PBS containing 0.01% Tween^®^ 20 and 0.5% BSA at pH 8 for 2 h, under gentle agitation, at room temperature. They were then rinsed three times with PBS for 10 min each, followed by incubation with the corresponding secondary antibodies (Table 2), conjugated with fluorochromes and diluted in PBS containing 0.01% Tween^®^ 20 and 1.5% BSA for 1 h, under light stirring at room temperature. Once more, they were rinsed three times with PBS for 10 min each. Samples of leukocytes with transferrin and VLPs were labeled with Phalloidin conjugated to AlexaFluor^®^ 594 and incubated for 20 min. Immediately after that, they were rinsed twice with PBS for 10 min each. Aliquots of the cell suspension from both items assayed were adhered to silanized slides and mounted with 5 µL Mowiol^®^ and coverslips. Samples were kept at 4 °C until CLSM analysis, using the Confocal Laser Scanning Microscope Zeiss 510 Meta of the Butantan Institute (FAPESP Process No. 2000/11624-5; Carl Zeiss GmbH, Jena, DEU).

## 5. Conclusions

HPV16 L1/L2 chimeric virus-like particles (VLPs) generated in this study demonstrated interactions with HEK 293-T cells in vitro and with peripheral blood mononuclear cells (PBMCs) ex vivo from healthy female volunteers. Both cell types internalized the VLPs. Our findings indicate that HPV16 L1/L2 chimeric VLPs can bind to the plasma membrane and be taken up by lymphocytes. Moreover, evidence suggests that these particles may exploit the clathrin-mediated endocytic pathway. This pathway—highly conserved among vertebrates and broadly distributed across the animal kingdom—together with other endocytic routes analyzed, supports the hypothesis that HPV16 L1/L2 chimeric VLPs do not require a specific receptor for lymphocyte internalization. Further studies are warranted to elucidate the precise molecular mechanisms underlying this process.

In this work, we likewise validated two CMV-driven mammalian expression vectors, pUF3/L1h (#874) and pUF3/L2h (#893), for the production of HPV L1 and L2 proteins in HEK 293-T and HEK 293-F cells. Both constructs exhibited robust protein expression, and co-transfection supported the assembly of VLPs closely resembling native HPV capsids. The inclusion of distinct purification and detection tags, along with complementary selection markers, enhances the versatility of these vectors for both fundamental virology research and translational applications.

Our findings underscore the potential of L1/L2 co-expression systems to advance next-generation HPV vaccine development, particularly those designed to broaden cross-type protection through incorporation of conserved L2 epitopes. Beyond vaccine research, the modular design of these vectors provides a flexible platform for studying immune modulation, epitope presentation, and therapeutic protein delivery. Collectively, this work establishes pUF3/L1h and pUF3/L2h as valuable tools for dissecting HPV biology and exploring innovative strategies to augment antiviral immunity.

This study, conducted with human peripheral blood leukocytes, sought to explore alternative pathways of HPV infection using VLPs produced through recombinant DNA technology. This approach enabled the generation of structurally defined HPV16 L1/L2 chimeric VLPs and provided a controlled system to investigate their interactions with immune cells ex vivo.

By focusing on leukocytes, we aimed to shed light on non-classical routes of HPV entry beyond the well-established epithelial tropism. Such investigations are crucial to understanding whether circulating immune cells may act as reservoirs, carriers, or modulators of viral dissemination. Advanced methodologies—including confocal microscopy, flow cytometry, molecular analyses, and transmission electron microscopy—allowed precise visualization of VLP crosstalk and colocalization with cellular markers, strengthening the evidence for alternative infection pathways. These findings contribute to a broader understanding of HPV biology, highlighting the possibility that leukocytes may play a role in viral persistence or immune evasion. Moreover, the recombinant production of chimeric VLPs underscores the versatility of molecular engineering, not only for mechanistic studies but also for translational applications in vaccine development. Ultimately, this research expands the horizon of HPV investigation, offering new perspectives on viral pathogenesis and potential strategies for prevention.

## Figures and Tables

**Figure 1:**
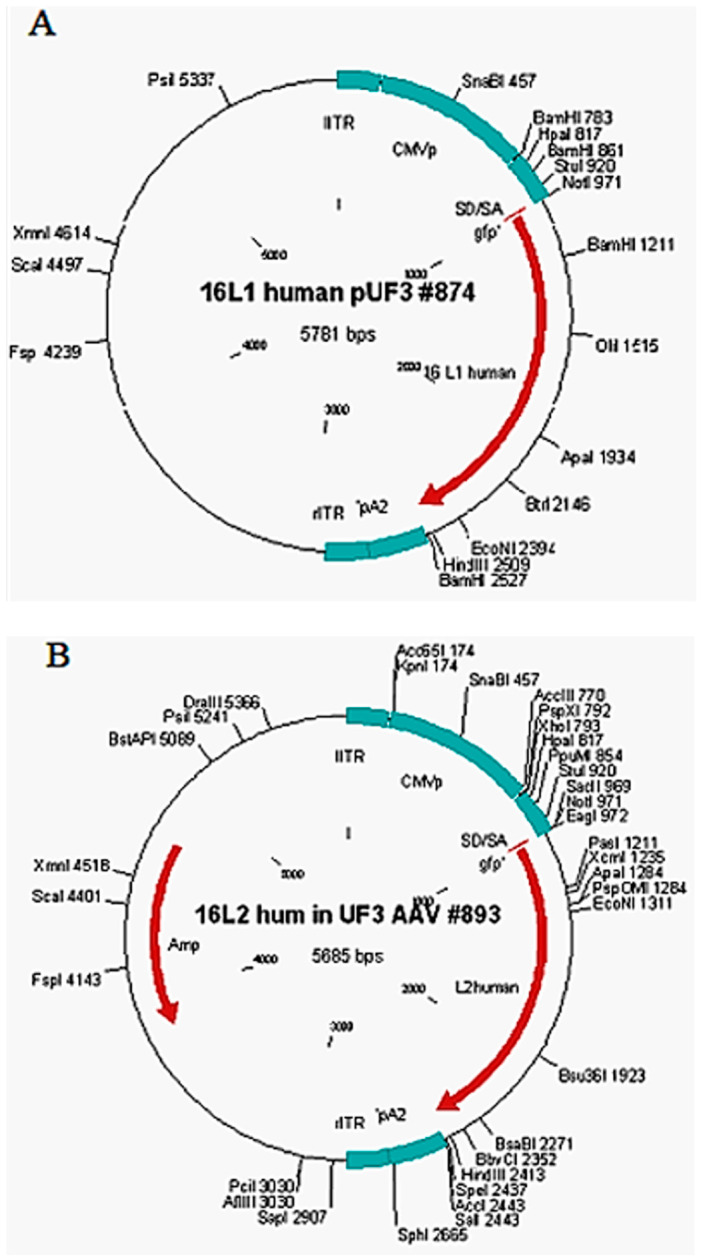
Design of CMV-driven expression vectors pUF3/L1h (#874—A) and pUF3/L2h (#893—B)—both optimized for heterologous protein expression in HEK 293T and 293F cells. Credit: Leder C et al., 2001 [14]. (**A**) pUF3/L1h (#874) contains the *HPV L1* gene downstream of the CMV immediate-early promoter and Kozak sequence, fused to an N-terminal His_6_-tag for purification. The vector includes a neomycin resistance cassette for selection, an SV40 polyadenylation signal for transcript stabilization, and a ColE1 origin for bacterial propagation [14]. (**B**) pUF3/L2h (#893) carries the *HPV L2* gene under CMV promoter control, with a C-terminal FLAG-tag for detection. The plasmid features a puromycin resistance marker, a BGH poly(A) signal, and a ColE1 origin. Restriction sites (EcoRI, BamHI, HindIII, XhoI, NotI for pUF3/L1h; NheI, XbaI, EcoRI, XhoI, NotI for pUF3/L2h) facilitate cloning and verification. Both constructs are optimized for high-level expression of HPV capsid proteins and can be co-transfected to study virus-like particle (VLP) assembly and immunogenicity [14].

**Figure 2:**
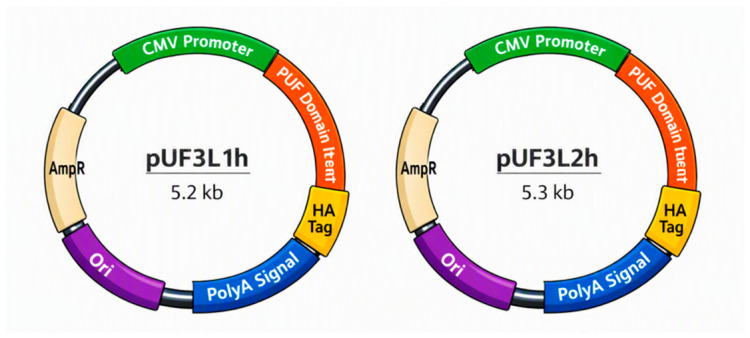
Schematic plasmid maps depict the architecture of two mammalian expression constructs engineered for PUF DNA-binding protein studies. Both vectors contain a PUF domain cloning site, HA epitope tag, and polyadenylation signal (PolyA) downstream of the promoter, as well as bacterial propagation elements (AmpR and Ori). The vectors pUF3L1h and pUF3L2h utilizes the CMV promoter, enabling strong but transient expression, providing stable, constitutive expression across diverse cell types. These complementary designs allow flexible modulation of PUF protein expression depending on experimental requirements.

**Figure 3:**
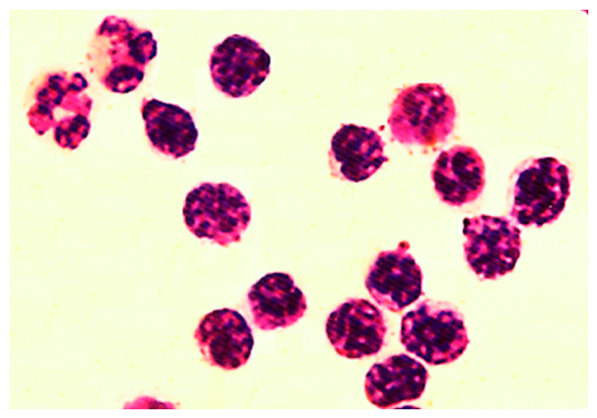
Human leukocytes isolated from peripheral blood and stained with May–Grünwald–Giemsa stain. The preparation demonstrates a mixed leukocyte pool, reflecting the yield of the separation method. Polymorphonuclear cells are identifiable by their multilobed nuclei and pale cytoplasm with fine granules. Lymphocytes by their dense basophilic nuclei and scant cytoplasm, and monocytes by their kidney-shaped nuclei and abundant gray-blue cytoplasm. The staining highlights nuclear detail and cytoplasmic contrast, facilitating morphological distinction among cell types. Image acquired using a 100× oil immersion objective (NA 1.2).

**Figure 4:**
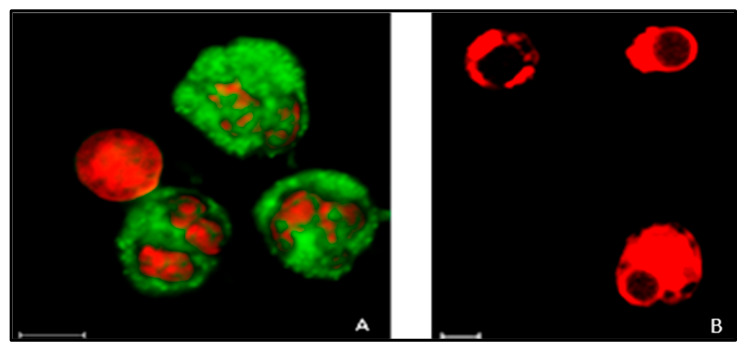
Cell Viability. Leukocyte cells obtained from the separation of human peripheral blood. In (**A**), nuclear labeling with PI (red) delimits cellular DNA, and DiOC6(3) (green) marks the membranes. In (**B**), leukocytes are labeled with Rhodamine 123 (red). CLSM Zeiss LSM 510 Meta. Magnification: 63× C-Apochromatic objective /1.4 Oil. Scale bars = 5 µm.

**Figure 5:**
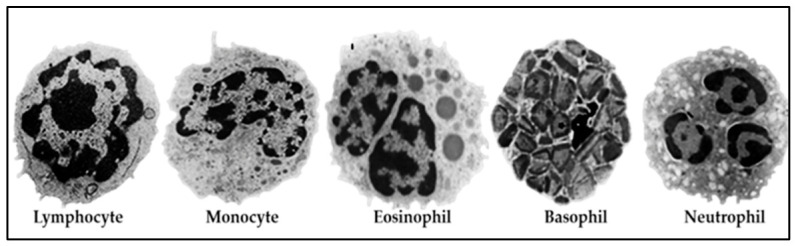
Electron micrographs of ultrathin sections of human leukocytes. Cells obtained by the peripheral blood leukocyte separation method. Conventional processing for transmission electron microscopy. Staining with lead citrate and uranyl acetate. Images acquired using a Zeiss EM 109 TEM operated at 80 kV with digital camera Mega View III and iTEM Basic Version 8.3.5. software, Olympus Soft Imaging Solutions GmbH, Olympus, Germany, at original magnifications of 7000×.

**Figure 6:**
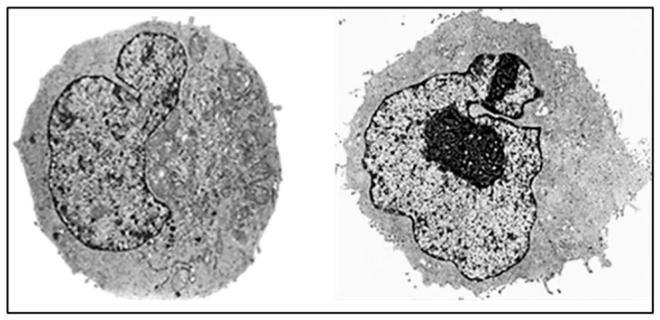
Ultrathin section electron micrographs of HEK 293-T (**left**) and HEK 293-F (**right**) cells, both derived from human kidney epithelium. Samples were conventionally processed for transmission electron microscopy and stained with lead citrate and uranyl acetate. Prominent cytoplasmic structures include lysosomes, mitochondria, the Golgi apparatus, the endoplasmic reticulum, as well as the nucleus and nucleolus. Images acquired using a Zeiss EM 109 TEM operated at 80 kV with digital camera Mega View III and iTEM Basic Version software, Olympus Soft Imaging Solutions GmbH, Olympus, Germany, at original magnifications of 7000×.

**Figure 7:**
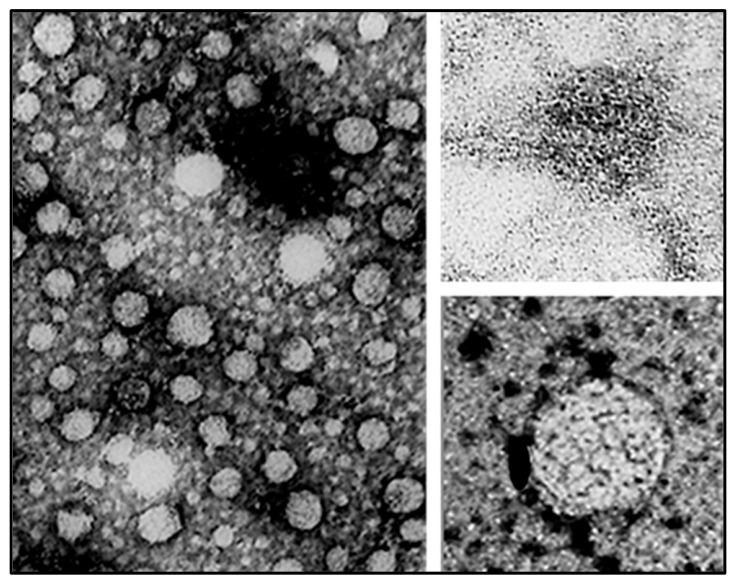
Transmission Electron Microscopy (TEM) of HPV16 L1/L2 chimeric virus-like particles (VLPs). Negative staining reveals structured HPV16 L1/L2 VLPs and particles undergoing self-assembly through the integration of L1 pentamers joined by L2, which confers enhanced structural stability. The right panel shows immunolabeling with anti-L1 antibody conjugated to 10 nm colloidal gold particles and anti-L2 antibody conjugated to 20 nm particles, highlighting the initial stage of HPV16 L1/L2 chimeric VLP formation. The bottom right image depicts an apparently complete HPV16 L1/L2 chimeric VLP, approximately 50 nm in diameter, obtained under identical conditions by Zeiss EM 109 TEM, 80 kV with digital camera Mega View III and iTEM Basic Version software, Olympus Soft Imaging Solutions GmbH, Olympus, Germany, at original magnifications of 20,000× (**left**) and 30,000× (**right**).

**Figure 8:**
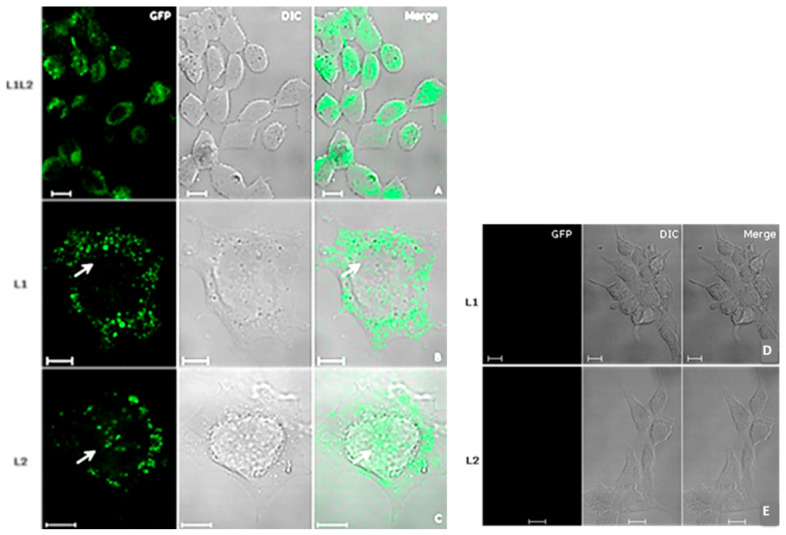
Expression of HPV16 L1 and L2 proteins in HEK 293-T cells transfected with pUF3/L1h and pUF3/L2h vectors, detected by fusion GFP fluorescence (left side panel). (**A**) Co-expression of L1-GFP and L2-GFP (green). (**B**) Expression of L1-GFP (green). (**C**) Expression of L2-GFP (green), localized in the nucleus (white arrows) and cytoplasm. Control assays for detection of L1-GFP and L2-GFP protein expression in HEK 293-T cells transfected with the empty vector (right side panel). (**D**) the L1-GFP control (green) and (**E**) the control (green) L2-GFP protein. Images acquired using the LSM 510 Meta Zeiss confocal microscope, magnification 63× C-Apochromatic objective/1.4 Oil. Scale bars = 10 µm.

**Figure 9:**
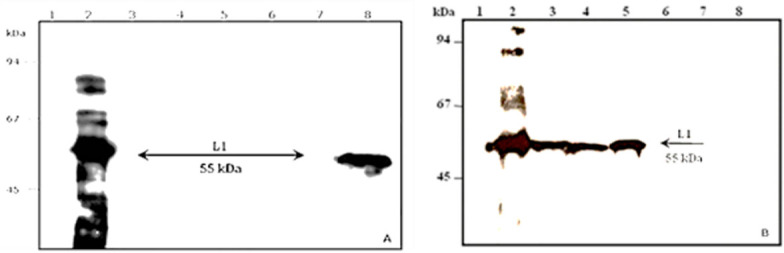
Expression of HPV16 L1 protein in HEK 293-T cells. Total extracts of HEK 293-T ((**A**)—channel 8; (**B**)—channels 3, 4 and 5) were analyzed by Western blot with the L1-specific antibody Camvir-1. The 55 kDa bands, indicated by the arrows, correspond to L1. No immunoreactive bands were detected in the CHO extracts ((**A**)—channels 3 and 5; (**B**)—channel 6). HPV 16 L1 VLPs produced in the fungi *Pichia pastoris* were used as positive controls (**A**,**B**—channel 2). Non-transfected cell lines ((**A**)—channel 4; (**B**)—channel 7) and cell lines transfected with the empty vector ((**A**)—channels 6 and 7; (**B**)—channel 8) were used as negative controls. Channel 1 (**A**,**B**) corresponds to the molecular mass marker.

**Figure 10:**
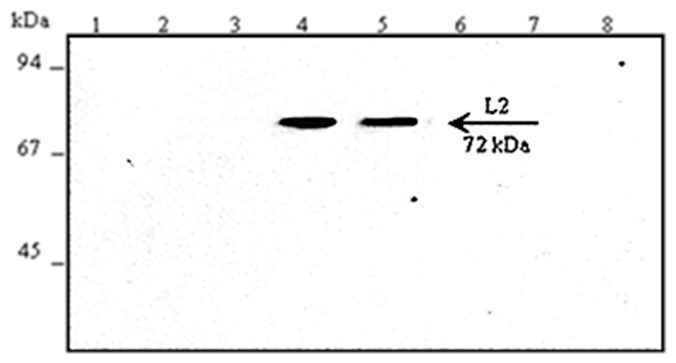
Expression of HPV16 L2 protein in HEK 293-T cells. Total extracts of HEK 293-T (channels 4 and 5) were analyzed by Western blot with an antibody specific to HPV 16 L2. The 72 kDa bands, indicated by the arrow, correspond to L2. Non-transfected cell lines (channels 2 and 3) and cell lines transfected with the empty vector (channels 6, 7 and 8) were used as negative controls. Channel 1 corresponds to the molecular mass marker.

**Figure 11:**
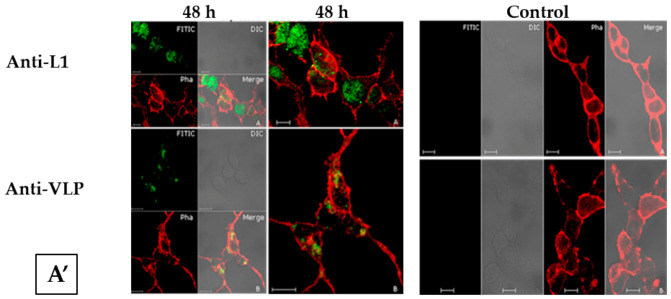

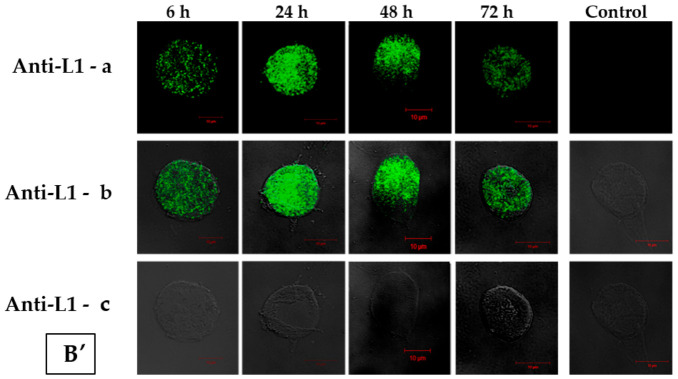
Detection of HPV16 L1 and VLPs produced by HEK 293-T (**A’**) and HEK 293-F (**B’**) cells using indirect immunofluorescence.

**Figure 12:**
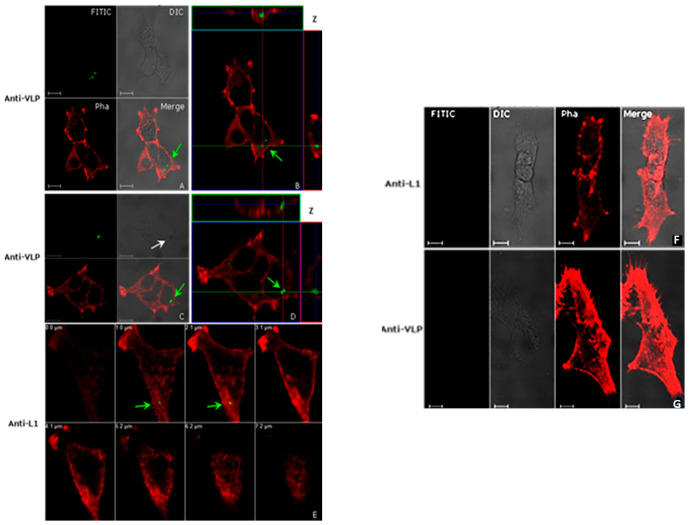
Crosstalk of the non-transfected HEK 293-T cell line with HPV16 VLPs, detected by indirect immunofluorescence. Cells were incubated with VLPs for 4 h and fixed with 2% PFA in PBS. In (**E**), the cells were immunolabeled with anti-L1 antibody, and in (**A**–**D**) with conformational anti-VLP antibody, both for HPV16, and revealed with the secondary antibody conjugated to FITC (green). The actin cytoskeleton was detected by Phalloidin conjugated with AlexaFluor^®^ 594 (red). VLP internalizations (green arrows) are shown in images (**B**) and (**D**), and L1 in (**E**), through Z-axis scan sections. (**B**) Sections 11.38 µm thick, with each section being 1.03 µm; (**D**) 9.67 µm thick sections, each section being 1.61 µm. In (**C**), a structure similar to an endocytic vesicle is observed (white arrow). Control assays of the interactions of non-transfected HEK 293-T cells with HPV16 VLPs, detected by immunofluorescence. In (**F**), the cells were treated with anti-L1 antibody, and in (**G**) with conformational anti-VLP antibody, both for HPV16, and revealed with the secondary antibody conjugated to FITC (green). The actin cytoskeleton was detected by Phalloidin conjugated with AlexaFluor^®^ 594 (red). CLSM Zeiss LSM 510 Meta. Magnification: 63× C-Apochromatic objective /1.4 Oil. Scale bar = 10 µm.

**Figure 13:**
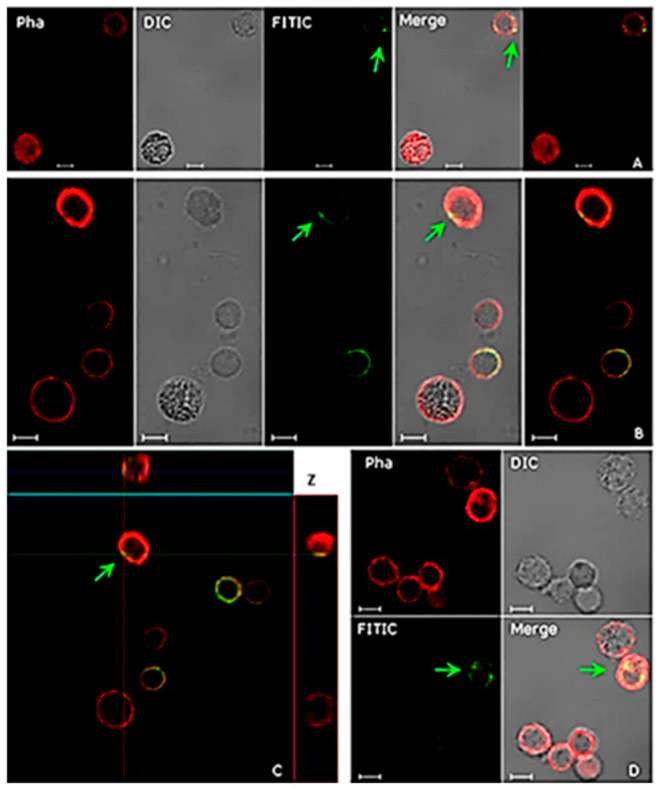
Crosstalk of human leukocytes with HPV16 virus-like particles (VLPs), visualized by indirect immunofluorescence. Leukocytes were incubated with HPV16 VLPs for 4 h, fixed, and immunostained with an anti-HPV16 L1 antibody, followed by a FITC-conjugated secondary antibody (green). The actin cytoskeleton was counterstained with AlexaFluor^®^ 594-conjugated phalloidin (red). Panels (**A**,**B**,**D**) (merged images) show the colocalization of VLPs with filamentous actin. Panel (**C**) illustrates VLP internalization (green arrows), demonstrated by Z-axis optical sections (9.50 µm total thickness, 1.60 µm per section). Images were acquired using CLSM Zeiss LSM 510 Meta with a 63× C-Apochromat/1.4 oil objective. Scale bar = 5 µm.

**Figure 14:**
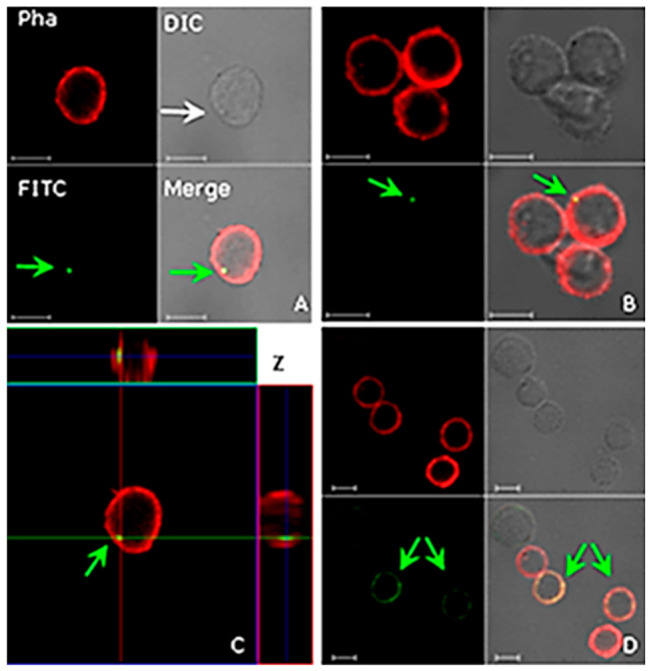
Crosstalk of human leukocytes with HPV16 virus-like particles (VLPs), visualized by indirect immunofluorescence. Leukocytes were incubated with HPV16 VLPs for 4 h, fixed, and immunostained with a conformational anti-HPV16 VLP antibody, followed by a FITC-conjugated secondary antibody (green). The actin cytoskeleton was counterstained with AlexaFluor^®^ 594-conjugated phalloidin (red). Panels (**A**,**B**,**D**) (merged images) show colocalization of VLPs with filamentous actin. Panel (**C**) demonstrates VLP internalization (green arrows), revealed by Z-axis optical sections (7.80 µm total thickness, 1.30 µm per section). In panel (**A**), a structure resembling an endocytic vesicle is indicated (white arrow). Images were acquired using CLSM Zeiss LSM 510 Meta with a 63× C-Apochromat/1.4 oil objective. Scale bar = 5 µm.

**Figure 15:**
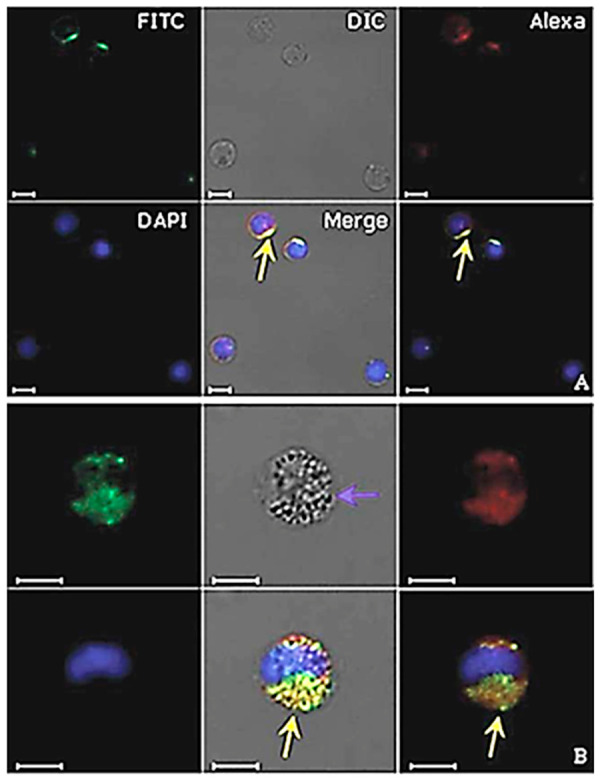
Leukocyte–HPV16 VLP interactions after 4 h of incubation, visualized by indirect immunofluorescence. Panel (**A**) shows that colocalization of HPV16 L1 and L2 proteins was detected using anti-L1 (green) and anti-L2 (red) antibodies, as expected, given that VLPs are composed of both proteins. This colocalization is evident in the image overlays (yellow arrows). Panel (**B**) shows substantial VLP internalization by leukocytes compared with other cell types, with vacuole-like structures indicated (purple arrow). At least 15 fields were analyzed per experiment, each containing 4–10 cells. Control assays showed no immunostaining with either L1 or L2 antibodies alone, or with structured HPV16 L1/L2 VLPs. Images were acquired using CLSM Zeiss LSM 510 Meta with a 63× C-Apochromat/1.4 oil objective. Scale bar = 5 µm.

**Figure 16:**
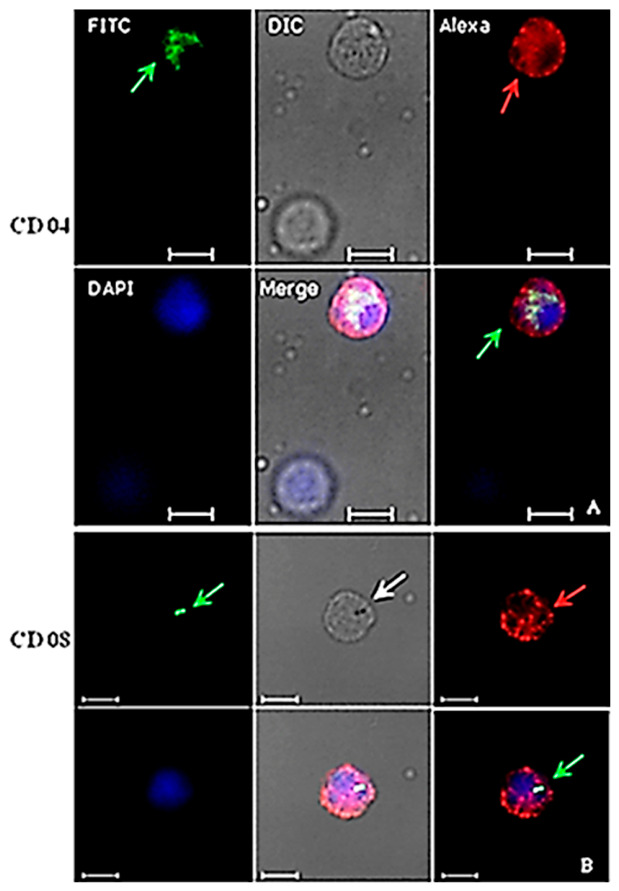
Identification of human leukocyte interactions with the HPV16 VLPs, analyzed by indirect immunofluorescence after 4 h of incubation. After fixation, cells were immunoassayed with the conformational anti-HPV16 VLPs and revealed by a secondary antibody conjugated to FITC (green). Then, the cells were treated with anti-CD04 (**A**) and anti-CD08 (**B**) and stained with a secondary antibody, AlexaFluor^®^ 546 (red color and red arrows). VLP internalization (green arrows) is visible in the image overlay (merged images). In (**B**), a structure similar to an endocytic vesicle is detectable (white arrow). CLSM Zeiss LSM 510 Meta. Magnification: 63× C-Apochromatic objective /1.4 Oil. Scale bar = 5 µm.

**Figure 17:**
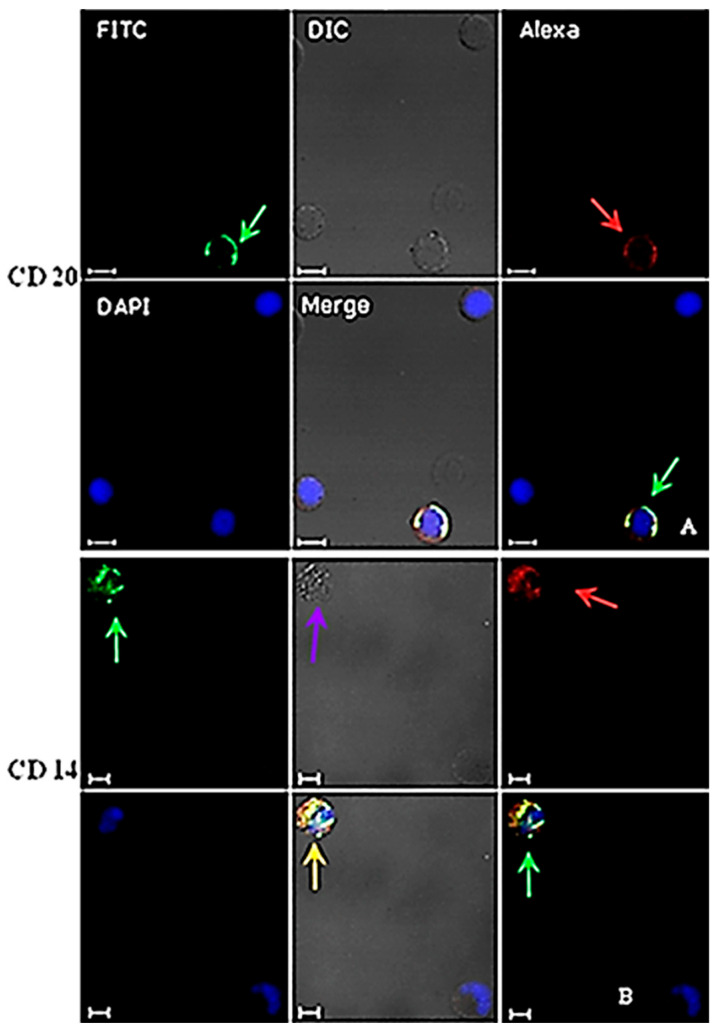
Identification of human leukocyte interactions with the HPV16 VLPs, analyzed by indirect immunofluorescence after 4 h of incubation. After fixation, cells were immunoassayed with the conformational anti-HPV16 VLPs and revealed by a secondary antibody conjugated to FITC (green). In (**A**) the treatment of cells with anti-CD20 and anti-CD14, followed by staining with a secondary antibody, AlexaFluor^®^ 546 (red color and red arrows), made VLP internalization (green arrows) visible in the image overlay (merged images). In (**B**), we can observe the colocalization of CD14 and VLPs (yellow arrow) and vacuole-like structures (purple arrow). CLSM Zeiss LSM 510 Meta. Magnification: 63× C-Apochromatic objective /1.4 Oil. Scale bar = 5 µm.

**Figure 18:**
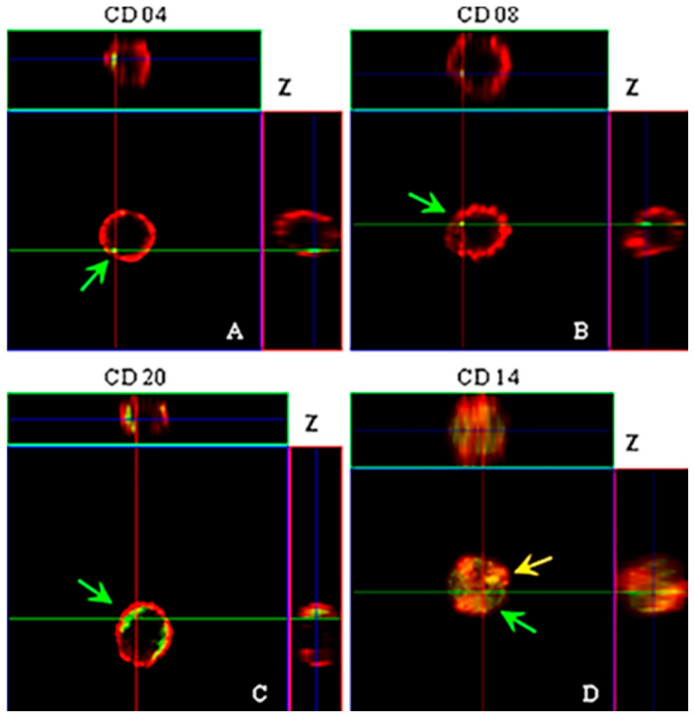
Identification of human leukocyte interactions with the HPV16 VLPs, analyzed by indirect immunofluorescence after 4 h of incubation. After fixation, cells were immunoassayed with the conformational anti-HPV16 VLPs and revealed by a secondary antibody conjugated to FITC (green). Then, the cells were treated with anti-CD20 (**C**), anti-CD04 (**A**), anti-CD08 (**B**), anti-CD14 (**D**) and AlexaFluor^®^ 546, revealed by a secondary antibody (red). VLP internalization (green arrows) is shown in the images through Z-axis sweep cuts. (**A**) 9.25 µm thick sections, each section measuring 0.84 µm; (**B**) 8.56 µm thick sections, each section measuring 0.95 µm; (**C**) 9.27 µm thick sections, each section measuring 1.03 µm; (**D**) 10,94 µm thick sections, each section measuring 1.40 µm. CLSM Zeiss LSM 510 Meta. Magnification: 63× C-Apochromatic objective/1.4 Oil. Scale bar = 5 µm.

**Figure 19:**
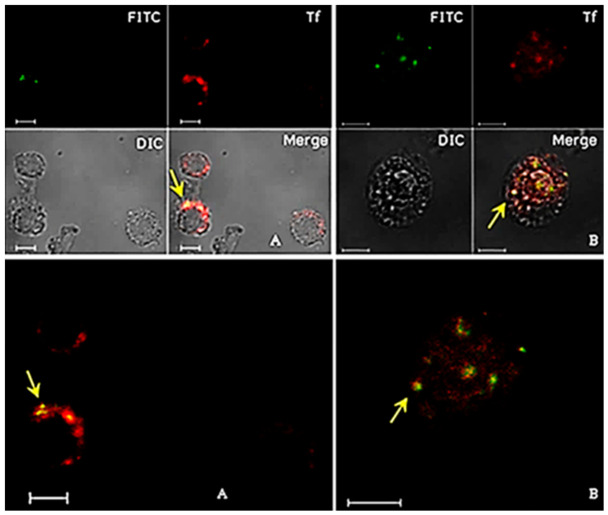
Colocalization of HPV16 VLPs and Tf in human white blood cells, detected by indirect immunofluorescence after 45 min of incubation. The leukocyte cells were incubated with the VLPs and the conjugated Tf TexasRed^®^ (red). Leukocytes were immunoassayed with an anti-HPV16 VLP antibody for 45 min and revealed by a secondary antibody conjugated to FITC (green). Panels (**A**) and (**B**) in confocal merge reveal colocalization of VLPs and Tf (yellow arrows). CLSM Zeiss LSM 510 Meta. Magnification: 63× C-Apochromatic objective /1.4 Oil. Scale bar = 5 µm.

**Figure 20:**
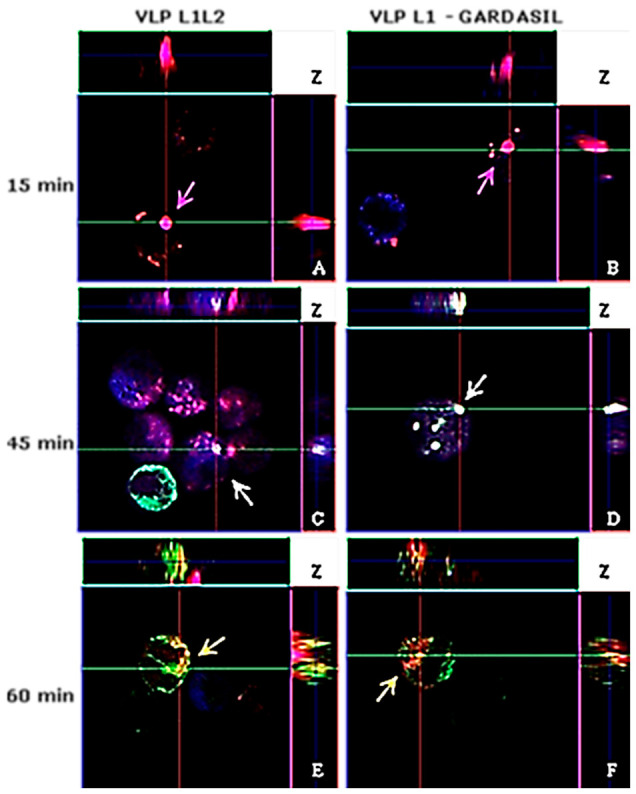
Three-dimensional imaging was used to analyze leukocyte interactions with virus-like particles (VLPs) produced in this study and with VLPs from the Gardasil vaccine. Interactions with transferrin (Tf) and transferrin receptor (TfR) were detected by indirect immunofluorescence. Leukocytes were incubated with VLPs and conjugated Tf-TexasRed^®^ (red). After fixation, cells were immunostained with an anti-HPV16 VLP antibody, revealed by a FITC-conjugated secondary antibody (green), and then treated with anti-CD71, revealed by AlexaFluor^®^ 633 (blue). CLSM Zeiss LSM 510 Meta. Magnification: 63× C-Apochromatic objective /1.4 Oil. Scale bar = 5 µm. Panels (**A**,**B**): colocalization of Tf and TfR (purple arrows). Panels (**C**,**D**): colocalization of VLP/Tf/TfR (white arrows). Panels (**E**,**F**): weak colocalization of VLP/Tf (yellow arrows).

**Figure 21:**
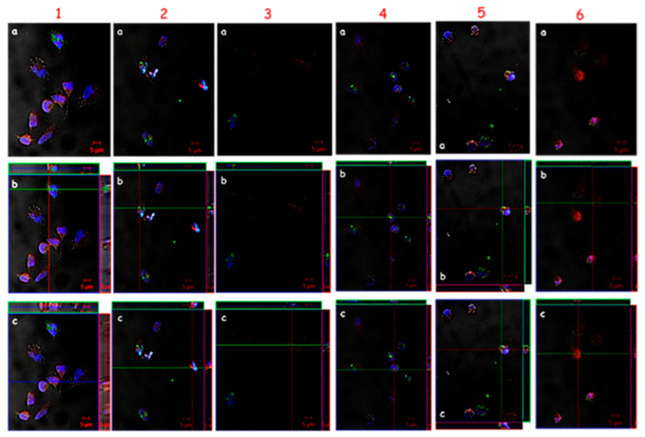
(1–6) Immunofluorescence assay detecting HPV VLP entry in PBMC, even in the presence of specific biochemical inhibitors of ligand uptake: (1) Sodium azide; (2) Chlorpromazine; (3) Liquemine; (4) rCTB; (5) Filipin; (6) Nystatin. (**a**) The overlay (merge) of images detected by the stain of L1 protein (green), L2 protein (red), and nuclei (blue); (**b**) Z-axis showing L1 protein within cells; (**c**) Z-axis showing that the entry of L2 protein in cells was not inhibited. CLSM Zeiss LSM 510 Meta. Magnification: 63× C-Apochromatic objective /1.4 Oil. Scale bar = 5 µm.

**Figure 22:**
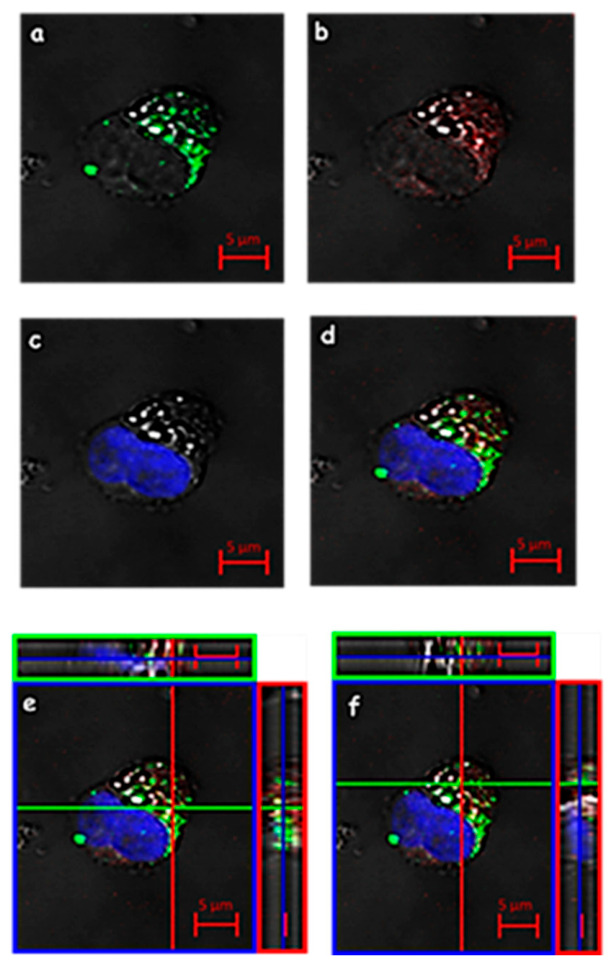
Assay reached with all inhibitors used in Figure 21 (1–6) in association. (**a**) Detection of L1 protein (green); (**b**) L2 protein within cells (red); (**c**) nuclei (blue); (**d**) the overlay (merge) of images (**a**–**c**); Z-axis showing the L1 (**e**) and L2 (**f**) proteins within cells. CLSM Zeiss LSM 510 Meta. Magnification: 63× C-Apochromatic objective/1.4 Oil. Scale bar = 5 µm.

**Figure 23:**
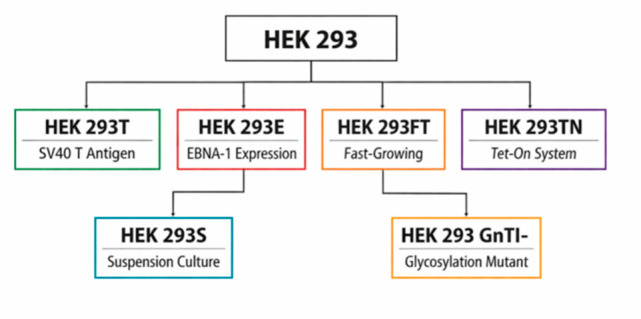
Schematic representation of the HEK 293 cell line family. The parental HEK 293 cell line is shown at the top, with arrows indicating the derivation of commonly used variants. Each derivative is highlighted with a distinct border color and labeled with its defining characteristic: HEK 293-T (SV40 large T antigen expression), HEK 293-FT (fast-growing phenotype), HEK 293-E (EBNA-1 expression), HEK 293T-N (Tet-On system), HEK 293-S (adapted to suspension culture), and HEK 293-GnTI (glycosylation mutant). This schematic illustrates the lineage and functional diversity of HEK 293 derivatives widely employed in molecular biology and biotechnology [80,82,83,84].

**Table 1:** List of primary antibodies used in immunocytochemistry reactions.

Antibody	Dilution	Description	Supplier
**Anti-L1 Camvir-1**	1:500	anti-mouse IgG_2a_, specific for HPV16	BD Biosciences
**Anti-L1 conformational**	1:100	anti-mouse IgG_2a_, specific for HPV16	Biodesign
**Anti-L2**	1:100	anti-rabbit IgG	Roden, R. MD/USA
**Transferrin conjugated**	1:60	TexasRed^®^ (Ex. 595 nm, Em. 615 nm)	Molecular Probes™
**Anti-Tf**	1:750	anti- rabbit IgG	Dako
**Anti-Ferritin**	1:750	anti- rabbit IgG	Dako
**Anti-CD71**	1:750	anti-mouse IgG_1_; Tf receptor	Dako
**Anti-CD04**	1:20	anti-mouse IgG_2a_; recognizes T lymphocytes “helper”	CALTAG™ Laboratories
**Anti-CD08**	1:20	anti-mouse IgG_2a_; recognizes cytotoxic T lymphocytes	CALTAG™ Laboratories
**Anti-CD14**	1:20	anti-mouse IgG_2a_; recognizes monocytes	CALTAG™ Laboratories
**Anti-CD20**	1:20	anti-mouse IgG_3_; recognizes B lymphocytes	CALTAG™ Laboratories
**Anti-L2**	1:100	anti-mouse HPV16 L2 (1–40 aa) monoclonal	Santa Cruz Biotechnology

**Table 2:** List of secondary antibodies used in immunocytochemistry reactions.

Antibody	Dilution	Description	Ex/Em. (nm)	Supplier
**FITC**	1:250	anti-mouse IgG_2a_	495/519	BD Biosciences
**AlexaFluor^®^546**	1:500	anti-mouse IgG	556/573	Molecular Probes™
**AlexaFluor^®^555**	1:500	anti-rabbit IgG	555/565	Molecular Probes™
**AlexaFluor^®^594**	1:500	anti-mouse IgG_3_	590/617	Molecular Probes™
**AlexaFluor^®^633**	1:500	anti-mouse IgG	632/647	Molecular Probes™
**AlexaFluor^®^633**	1:500	anti-rabbit IgG	632/647	Molecular Probes™

**Table 3:** Biochemical inhibitors used to examine their effects on the entry of HPV VLPs in PBMC.

Inhibitors	Inhibition
**Chlorpromazine**	Clathrin pathway
**rCTB (Clostridium toxin B)**	Actin-dependent cells process
**Filipin and Nystatin**	Caveolae pathway
**Liquemine**	HSPG pathway
**Sodium azide**	Endocytic pathways

**Table 4:** Interactions of HPV16 VLPs produced in this study with PBMC (mononuclear cells) from the peripheral blood of healthy female volunteers.

PBMC	Receptor	Percentage of Cells That Interacted with VLPs ^1^
Lymphocyte T	CD04	52% ± 3
Lymphocyte T	CD08	47% ± 2
Lymphocyte B	CD20	48% ± 3
Monocyte	CD14	23% ± 5

^1^ The percentage of cells that interact with HPV16 VLPs was calculated by the number of cells recognized by anti-CD antibodies that have internalized the particles. The result corresponds to the analysis in duplicate and is representative of at least four trials.

## Data Availability

The original contributions presented in this study are included in the article. Further inquiries can be directed to the corresponding author.

## Abbreviations

This manuscript contains the following abbreviations:

HPV	Human Papillomavirus
VLP	Virus Like Particles
L1	Major Capsid Protein
L2	Minor Capsid Protein
HEK293-T	Human Epithelial Kidney Cell Lineage Grown Attached
HEK293-F	Human Epithelial Kidney Cell Lineage Grown in Suspension
CD71	Transferrin Receptor
CD4	T Lymphocyte Membrane Receptor
CD8	T Lymphocyte Membrane Receptor
CD20	B Lymphocyte Membrane Receptor
CD14	Monocyte Membrane Receptor
rCTB	Recombinant Cholera Toxin B Subunit is the Non-Toxic Portion
CT	Cholera Toxin
HIV	Human Immunodeficiency Virus
CMV	Cytomegalovirus
PsV	Pseudovirus
pUF3L1h Vector	Humanized Expression Vector for L1 Protein
pUF3L2h Vector	Humanized Expression Vector for L2 Protein
EDTA	Anticoagulant
Tf	Transferrin
DMEM	Dulbecco’s Modified Medium
FBS	Fetal Bovine Serum
RPMI	Roswell Park Memorial Institute Cell Culture Medium
PBMC	Peripheral Blood Mononuclear Cell
BSA	Bovine Serum Albumin
TEM	Transmission Electron Microscopy
DHFR	Dihydrofolate Reductase
GS	Glutamine Synthase
VIA	Visual Inspection with Acetic Acid
TIME	Tumor Immune Microenvironment
